# FGF4 activates FGFR1 - PI3K/AKT signaling to enhance Clec10a-mediated intracellular myelin debris processing and promote spinal cord repair

**DOI:** 10.1186/s12974-026-03743-0

**Published:** 2026-02-23

**Authors:** Wenjie Lu, Minghao Jiang, Junyu Zhuang, Jiahui Song, Cheng Zhou, Yangbo Zhou, Zhongwei Zhu, Aimin Wu, Sunren Sheng, Sipin Zhu, Zhouguang Wang

**Affiliations:** 1 Department of Orthopaedics, The Second Affiliated Hospital and Yuying Children’s Hospital of Wenzhou Medical University, Wenzhou Medical University, Wenzhou, Zhejiang 325000 China; 2 The First Affiliated Hospital of Wenzhou Medical University, Wenzhou Medical University, Wenzhou, Zhejiang 325035 China; 3 State Key Laboratory of Macromolecular Drugs and Large-scale Preparation, School of Pharmaceutical Science, Wenzhou Medical University, Wenzhou, Zhejiang 325035 China; 4 Oujiang Laboratory (Zhejiang Lab for Regenerative Medicine, Vision and Brain Health), School of Pharmaceutical Science, Wenzhou Medical University, Wenzhou, Zhejiang 325000 China

**Keywords:** FGF4, Clec10a, Myelin debris, Phagocytosis, PI3K/AKT signaling, Endosomal-Lysosome

## Abstract

Myelin debris accumulation after spinal cord injury (SCI) drives persistent neuroinflammation, lysosomal dysfunction, and lipid overload in macrophages, ultimately impairing tissue repair. Here, we identify fibroblast growth factor 4 (FGF4) as a previously unrecognized regulator of macrophage-mediated myelin debris clearance. Endogenous FGF4 transiently increased in the early phase of SCI but rapidly declined. Using in vitro models, we demonstrate that exogenous FGF4 markedly enhances myelin debris phagocytosis through activation of the FGFR1-PI3K/AKT signaling pathway, leading to upregulation of Clec10a, a C-type lectin receptor not previously linked to myelin debris processing. Silencing Clec10a significantly mitigated the phagocytic and neuroprotective benefits of FGF4, supporting Clec10a as an important mediator of this response. D-GalNAc competitive inhibition assays showed that Clec10a does not rely on the conserved carbohydrate-recognition domain to bind to myelin debris. FGF4 enhanced the maturation and degradative efficiency of the endolysosomal system, driving internalized myelin debris through Rab5^+^ early endosomes, Rab7^+^ late endosomes, and Lamp1^+^ lysosomes, where CTSD-mediated proteolysis was restored. This correction of lysosomal dysfunction was accompanied by reduced lysosomal membrane permeabilization. In vivo administration of FGF4 enhanced myelin debris clearance and alleviated lipid accumulation at the injury site, accompanied by attenuation of NLRP3 inflammasome activity and a shift in macrophage phenotypes toward a reparative state. Together, our results identify FGF4 as a previously unrecognized regulator of macrophage phagosome maturation and lysosomal function after spinal cord injury, linking FGFR1–PI3K/AKT signaling to Clec10a-mediated intracellular processing of internalized myelin debris and inflammation resolution.

**Supplementary Information:**

The online version contains supplementary material available at 10.1186/s12974-026-03743-0.

## Background

Spinal cord injury (SCI) is a severe degenerative disease that causes varying degrees of impairment in sensory, motor and autonomic nerve functions [1, 2]. The social and economic burden caused by the loss of these functions has increased [3]. However, the development of effective treatment plans after SCI is very limited. Common methods such as reducing spinal cord edema and neurotrophic drugs have limited efficacy [4, 5]. Therefore, it is particularly important to fully study the pathophysiological mechanisms after spinal cord injury and develop effective therapeutic targets to improve treatment.

Spinal cord injury causes immediate mechanical damage, followed by a long-term secondary cascade reaction, including hemorrhage, ischemia, oxidative stress, excitotoxicity and neuroinflammation [6]. A clear characteristic of this stage is the accumulation of extensive demyelination and lipid-rich myelin debris in the lesion area [7]. The myelin debris contain molecules related to axonal growth inhibition, such as Nogo-A, oligodendrocyte myelin glycoprotein (OMgp). And the debris act as an inflammatory stimulus, thereby exacerbating the unfavorable post-injury microenvironment and limiting repair [8, 9]. It is noteworthy that although phagocytes are rapidly recruited, the remnants of myelin debris often persist for several weeks, indicating that endogenous clearance is insufficient to meet the continuous demand for debris decomposition [10].

Therefore, enhancing the clearance of myelin debris has become a feasible strategy for improving the post-injury environment, supporting axon regeneration, and promoting functional recovery. Multiple studies have shown that specific innate receptors and immune metabolic programs determine the efficiency of myelin debris internalization and processing. Macrophage scavenger receptors play a significant role in this process, for example, up-regulation of the collagen-bound scavenger receptor MARCO enhances the ability of macrophages to phagocyte myelin debris, and promotes axon regeneration and nerve function repair [11]. TLR4 signaling is related to myelin debris clearance, and pharmacological activation of TLR4 (such as the non-toxic vaccine adjuvant E6020) can accelerate myelin debris clearance and promote axon regeneration [12]. Additionally, complement C3, CD47, and inhibitory CD200R are all related to the clearance of myelin debris [13–15]. It is worth noting that scavenger receptors may have a dual nature. For instance, macrophage scavenger receptor 1 (MSR1) and scavenger receptor CD36 stimulates the phagocytosis of myelin debris along with the production of foamy macrophages and pro-inflammatory polarization after spinal cord injury, highlighting the necessity of combining myelin debris uptake with lipid processing [16, 17].

A primary challenge is that macrophages that ingest lipid-rich myelin debris easily undergo foam-cell transformation. Like atherosclerotic plaques, SCI lesions often have foamy phagocytes full of lipid droplets and cholesterol from myelin [18]. Increasing lipid export is becoming more and more recognized as an important part of effective clearance. In this context, ATP-binding cassette transporter A1 (ABCA1), a primary regulator of phospholipid and cholesterol efflux, is particularly significant, and strategies that enhance ABCA1-dependent efflux (such as with APOA-I peptidomimetic) demonstrate potential in improving lipid management and recovery in SCI-associated pathology [19]. Oxidized lipids from myelin can make lysosomes less stable, cause the lysosomal membrane to become more permeable, and turn on the NLRP3 inflammasome. This results in increased tissue inflammation and pyroptosis that is dependent on Caspase-1/GSDMD [20, 21].

Mechanistically, myelin debris degradation by macrophages relies heavily on the endosome-lysosome system. Internalized cargo traffics through Rab5^+^ early endosomes, then to Rab7^+^ late endosomes, and finally enter Lamp1^+^ lysosomes, where lysosomal proteases including cathepsin D (CTSD) are activated to achieve proteolysis [22, 23]. PI3K activity is a critical determinant of phagosome maturation, vesicular trafficking, and degradative capacity [24, 25]. Furthermore, some studies have also shown that during the pathological processes of the central nervous system related to aging, autophagy may also be utilized to degrade and remove myelin debris in microglia [26].

Fibroblast growth factors (FGFs) are pleiotropic ligands involved in tissue repair and immunometabolic regulation [27]. Among them, FGF4 is a canonical paracrine FGF with strong affinity for FGFR1-containing complexes and has been implicated in regenerative contexts, including neural injury related repair [28]. However, It is still not known if FGF4 directly changes macrophage programs for clearing myelin debris or how it connects phagocytosis with lysosomal homeostasis and lipid excretion. Given that macrophages express FGFRs and that PI3K/AKT signaling governs core steps of phagocytic processing [29, 30], identifying an FGF4–FGFR axis that engages PI3K/AKT to drive productive myelin debris clearance may provide both mechanistic insight and a therapeutic entry point.

In parallel, injury microenvironments may engage non-canonical phagocytic modules beyond classical scavenger receptors [31, 32]. Clec10a (macrophage galactose-type lectin; human ortholog CLEC10A) is a C-type lectin linked to endocytic trafficking and immunoregulation in myeloid cells [33], but its role in myelin debris processing after SCI remains poorly defined. In this study, we hypothesized that FGF4 promotes productive macrophage clearance of myelin debris via FGFR1–PI3K/AKT activation and induction of a Clec10a-mediated phagocytic program, thereby enhancing endolysosomal maturation, improving lipid handling and ultimately supporting neuronal protection and functional recovery after SCI.

## Materials and methods

### Materials

Antibodies from Santa Cruz Biotechnology (Dallas, TX, USA) included anti-Clec10a (sc-56109), anti-F4/80 (sc-377009), anti-CD86 (sc-28347), and anti-LAMP1 (sc-20011). Antibodies purchased from Affinity Biosciences (Cincinnati, OH, USA) comprised anti-MBP (AF4085), anti-Bcl-2 (AF6139), anti-cleaved caspase-3 (AF7022), anti-p-FGFR1 (AF3157), anti-p-PI3K (AF3241), anti-PI3K (AF6241), anti-CD206 (DF4149), anti-cleaved caspase-1 (AF4005), anti-SYP (BF0348), and anti-5-HT1A (AF5453). Cell Signaling Technology (Danvers, MA, USA) provided anti-Rab5 (46449 S), anti-Rab7 (9367 S) and anti-TMEM119 (98778). Primary antibodies from Proteintech (Wuhan, China) included anti-β-actin (20536-1-AP), anti-ABCA1 (26564-1-AP), anti-NLRP3 (30109-1-AP), anti-GSDMD (20770-1-AP), anti-CTSD (21327-1-AP), anti-NF200 (18934-1-AP), and anti-TH (25859-1-AP). From Abcam (Cambridge, UK), we used anti-βIII-tubulin (ab18207), anti-GFAP (ab4674), anti-NeuN (ab104224), and anti-MAP2 (ab5392). Antibodies purchased from ZenBio (Chengdu, China) included anti-p-AKT (341790), anti-AKT (342529), and anti-IL-1β (511369). Bioss (Beijing, China) supplied both anti-FGF4 antibodies (bs-1256R and bsm-51757 M). Antibodies purchased from BioLegend (San Diego, CA, USA) included anti-Ly-6 C (128001). Secondary antibodies were purchased from Abcam (Cambridge, UK), including Goat Anti-Chicken IgY H&L (Alexa Fluor 488, ab150173), Goat Anti-Rat IgG H&L (Alexa Fluor 488, ab150157), Donkey Anti-Rabbit IgG H&L (Alexa Fluor 647, ab150063), and Donkey Anti-Mouse IgG H&L conjugated to Alexa Fluor 488 (ab150109), Alexa Fluor 555 (ab150110), or Alexa Fluor 647 (ab150111). Recombinant proteins and small molecules, including FGF4 (HY-P7014), PD173074 (HY-10321), and N-acetyl-D-galactosamine (HY-128852), were procured from MedChemExpress (Monmouth Junction, NJ, USA). All materials were used according to the manufacturers’ instructions unless otherwise specified.

### Establishment of the mouse spinal cord injury model

Adult female C57BL/6 mice (20–25 g) were sedated with 1% pentobarbital sodium before operation. Following shaving and disinfection of the surgical site, a midline skin incision was executed, subsequently leading to the excision of the spinous processes and laminectomy at the T9–T10 level to reveal the dorsal aspect of the spinal cord. A moderate contusion injury was produced utilizing a pneumatic-electronic impactor (68099 II, RWD Life Science, Shenzhen, China) with defined impact parameters, including an impact velocity of 1.5 m/s, a displacement depth of 0.6 mm, and a dwell time of 0.5 s. The successful induction of spinal cord injury (SCI) was verified by the observation of central cord bleeding, a tail flick upon contact, and immediate total hindlimb paralysis following the injury. Postoperative treatment involved manual bladder expression bi-daily until spontaneous urination was reestablished. Sham-operated animals underwent anesthesia and laminectomy without contusion injury.

### FGF4 administration

FGF4 was injected intraperitoneally at a dose of 9 µg/kg. Recombinant FGF4 was freshly prepared in sterile saline immediately before use. Mice received FGF4 on a post-injury dosing schedule of 1, 3, 5, 7, 9, and 11 days after SCI, for a total of six injections. Control animals were injected with an equal volume of saline solution at the corresponding time points. All injections were performed under sterile conditions.

### Cell culture

Primary bone marrow-derived macrophages (BMDMs) were extracted from 6 to 8 week-old C57BL/6 mice. Post-euthanasia, mice were submerged in 75% ethanol for 5 min for surface sterilization. Both femurs and tibias were exposed under aseptic conditions, and the epiphyses were removed with sterile scissors. Bone marrow was flushed out using DMEM high-glucose medium supplemented with 10% fetal bovine serum (FBS), penicillin/streptomycin, and 20 ng/mL M-CSF (MCE, HY-P7085). The cell suspension was filtered through a 70-µm cell strainer, centrifuged, and treated with erythrocyte lysis for 5 min. Cells were resuspended in full macrophage media and subsequently plated in suitable culture plates. BMDMs were cultivated for 5 to 7 days, with medium changes every 2 days, to ensure complete differentiation before experimental application. For FGF4 stimulation, Cells were pretreated with FGF4 (10 ng/mL) for 2 h, subsequently followed by the addition of myelin debris (1 mg/mL). For FGFR1 inhibition, cells were pre-incubated with PD173074 (50 nM) for 1 h, after which FGF4 (10 ng/mL) was added for 2 h, and myelin debris (1 mg/mL) was subsequently introduced. For GalNAc competition experiments, cells were co-pretreated with N-acetyl-D-galactosamine (50 nM) and FGF4 (10 ng/mL) for 2 h before adding myelin debris (1 mg/mL).

Primary cortical neurons were prepared from neonatal C57BL/6 mice (postnatal day 0–1). The cerebral cortices were rapidly dissected in ice-cold HBSS under sterile conditions. After removal of the meninges, cortical tissue was dissected into small pieces and digested with 0.25% trypsin–EDTA at 37 °C for 10 min. Enzymatic digestion was stopped by adding DMEM/F12 containing 10% FBS, and the tissue was gently triturated to obtain a single-cell suspension. The cell suspension was passed through a 70 μm cell strainer and plated onto poly-D-lysine–coated culture dishes. After 12 h, the cultured neurons were replaced with Neurobasal basal medium supplemented with B27, GlutaMAX, and penicillin/streptomycin, with half of the medium being refreshed every 3 days. Experiments were performed on neurons between DIV (days in vitro) 7–10, when stable neuronal morphology and synaptic development were established.

### Myelin debris (MD) preparation and Dil labeling

Myelin debris was isolated from adult mouse brains using sucrose density–gradient ultracentrifugation. Brain tissue was homogenized in 0.32 M sucrose and carefully overlaid with 0.83 M sucrose. The homogenate was transferred into 13.2-mL polyallomer ultracentrifuge tubes (Beckman, 331372) and centrifuged at 100,000 × g for 40 min at 4 °C. The opaque myelin detritus layer situated at the 0.32/0.83 M sucrose interface was harvested, diluted in Tris–Cl buffer, and subjected to further washing through ultracentrifugation at 100,000 × g for 30 min under identical temperature conditions. The final particle was resuspended in PBS, and the concentration was calibrated to 100 mg/mL.

For fluorescent labeling, purified myelin debris was incubated with 5 µM Dil (Beyotime, C1036) for 10 min at room temperature, followed by centrifugation to remove excess dye. The labeled debris was washed twice with PBS, resuspended to its original concentration for immediate use.

### Timeline of BMDM pretreatment, myelin debris exposure, and readouts

BMDMs were pretreated with FGF4 for 2 h before myelin debris exposure. A schematic overview of the stimulation and analysis timeline is provided in Supplementary Fig. S1. Briefly, for binding assays, BMDMs were incubated with Dil-labeled myelin at 4 °C for 30 min. For early trafficking assays, cells were incubated at 37 °C and analyzed at 5, 15, 30, and 60 min and 6 h to assess colocalization of internalized Dil-myelin with Rab5, Rab7, Lamp1, and Clec10a. For longer-term uptake readouts, MBP⁺ area within F4/80⁺ cells was quantified at 6, 12, 18, and 24 h after myelin debris exposure. At 24 h, cells were harvested for Western blotting, qPCR, and Oil Red O staining.

### Oil red O staining

Oil Red O staining was performed using the Modified Oil Red O Staining Kit (Beyotime, C0158S). BMDMs grown in 12-well plates were washed with PBS and fixed in 4% paraformaldehyde for 15 min at room temperature. Cells were then incubated with Oil Red O working solution for 30 min, briefly rinsed with the supplied wash buffer, and washed with PBS to remove excess dye. After adding PBS to cover the cells, images were captured immediately using a bright-field microscope. Fresh frozen spinal cords were embedded in OCT and cut into 16 μm sections. Sections were washed with PBS and briefly rinsed with the supplied wash buffer, followed by incubation with Oil Red O working solution for 30 min at room temperature. Following staining, the working solution was discarded, and the washing solution was applied again for 5 s to remove excess dye. Slides were then immersed in distilled water on a shaker for 20 s to ensure complete removal of background staining. After rinsing, sections were mounted with an anti-fade aqueous mounting medium and imaged immediately using an inverted bright-field microscope.

### Hematoxylin and eosin (H&E) and luxol fast blue–cresyl violet myelin staining

H&E staining was conducted using a commercial kit (Solarbio, G1121). Sections were washed in PBS three times (5 min each), stained with hematoxylin for 1 min, rinsed in distilled water for 10 s, differentiated for 5 s, and washed for 20 s. Slides were blued for 30 s, and then with eosin for 30 s, rinsed briefly, dehydrated, cleared, and mounted for bright-field imaging. For myelin staining, sections were incubated overnight in Luxol fast blue solution (Solarbio, G3245) at room temperature, washed with 95% ethanol, and rinsed with water. Slides were differentiated for 5 s, followed by additional differentiation in 70% ethanol for 10–20 s until gray and white matter were clearly distinguished. Sections were then counterstained with Cresyl violet for 30 s, rinsed, dehydrated, cleared, and mounted.

### Immunofluorescence(IF) staining and quantitative analysis

Immunofluorescence staining was performed on both cultured cells and spinal cord cryosections. For tissue samples, cryosections were washed with PBS, followed by antigen retrieval using citrate antigen retrieval buffer (Beyotime, P0081). Cells or tissue sections were then washed with PBS and blocked with 5% BSA for 1 h at room temperature. Primary antibodies were diluted in 1% BSA and incubated with the samples overnight at 4 ℃. After washing with PBS, appropriate fluorophore-conjugated secondary antibodies were applied for 1 h at room temperature in the dark. Following final PBS washes, samples were mounted using an anti-fade mounting medium with DAPI (Beyotime P0131). Images were acquired using either an OLYMPUS VS200 digital slide scanning system or a Nikon laser scanning confocal microscope under identical exposure and acquisition settings.

Serial transverse sections were collected continuously along the spinal cord from the rostral to caudal direction and numbered sequentially. To minimize anatomical variability across animals, sections with the same or adjacent index number from different mice were used for each staining. For quantitative analyses, three transverse sections per mouse were systematically sampled at 200-µm intervals. Within each section, three randomly selected, non-overlapping fields were analyzed within predefined regions of interest (ROIs). Image acquisition parameters were kept constant across all groups. Quantitative analyses were performed using ImageJ, and all quantifications were conducted by investigators blinded to group allocation.

### Immunohistochemistry (IHC)

Briefly, Sections were rinsed with PBS and subjected to antigen retrieval using citrate buffer. After cooling, endogenous peroxidase activity was quenched with 3% H_2_O_2_, followed by blocking in 5% BSA for 1 h at room temperature. Sections were then incubated with primary antibodies diluted in 1% BSA overnight at 4 °C. After PBS washes, appropriate HRP-conjugated secondary antibodies were applied for 1 h at room temperature. Signal was developed using DAB reagent (Zhongshan Golden Bridge, ZLI-9017), followed by hematoxylin counterstaining. Sections were dehydrated, cleared, mounted, and imaged under a bright-field microscope.

### Western blot (WB)

Protein samples from cells or spinal cord tissues were extracted using RIPA lysis buffer (Beyotime, P0038) supplemented with protease and phosphatase inhibitors. For spinal cord injury tissues, lesion segments were homogenized using a tissue grinder at 60 Hz for 60 s prior to lysis. Lysates were incubated on ice for 30 min and subsequently centrifuged at 12,000 × g for 15 min at 4 °C to isolate the supernatant. Protein concentrations were quantified employing a Coomassie Brilliant Blue assay in accordance with the manufacturer’s guidelines. Equal quantities of protein were subjected to SDS–PAGE and subsequently deposited onto PVDF membranes. Membranes were incubated with QuickBlock Western Blocking Buffer (Beyotime, P0252) for 15 min at room temperature, followed by overnight incubation with primary antibodies at 4 °C. Following rinsing, the membranes were incubated with HRP-conjugated secondary antibodies for one hour at room temperature. Protein bands were detected utilizing the IQ800 imaging system (GE Healthcare, USA), and densitometric analysis was conducted with ImageJ software (NIH, Bethesda, MD, USA).

### Transmission electron microscopy (TEM) of spinal cord tissue

Ultrastructural analysis of macrophages in spinal cord injury lesions was performed using TEM. Lesion core tissue was fixed in 2.5% glutaraldehyde/0.1 M phosphate buffer (4 °C), post-fixed with 1% osmium tetroxide, dehydrated via ethanol gradients, and embedded in epoxy resin. Ultrathin sections were stained with uranyl acetate/lead citrate. Macrophages were identified by ultrastructural markers: phagocytosed myelin debris and lipid-rich inclusions.

### Lentiviral transduction of BMDMs

Lentiviral transduction was employed to silence Clec10a expression in BMDMs. Cells were inoculated at an appropriate density and transduced with either control lentivirus (LV-NC) or LV-shClec10a (1 × 10^8^ TU/mL, MOI = 20) in the presence of 10 µg/mL polybrene. BMDMs were exposed to the lentiviral particles for 12 h, following which the infection medium was substituted with fresh, complete medium. Cells were subsequently cultured for an additional 72 h to confirm sufficient knockdown efficiency before collection for downstream analyses.

### Conditioned medium preparation and neuronal treatment

To assess the impact of residual myelin debris remaining after macrophage phagocytosis on the survival of neurons and axonal growth, BMDMs were subjected to the indicated treatments for 24 h, and the culture supernatants were then collected. The supernatants were clarified by centrifugation at 1500 rpm for 5 min to remove the precipitate, thereby obtaining a culture medium without cells. The filtered conditioned medium was subsequently mixed with neuronal basal medium and applied to cultured neurons for 24 h. After incubation, neuronal apoptosis and axonal outgrowth were evaluated using the corresponding assays.

### Flow cytometric analysis of neuronal apoptosis after conditioned medium treatment

Neuronal apoptosis after exposure to conditioned medium from differently treated BMDMs was measured using an Annexin V–FITC/PI apoptosis kit (Beyotime, C1062M). After 24 h of treatment, neurons were washed with PBS, dissociated into single cells, and resuspended in Annexin V binding buffer. Annexin V–FITC and PI were then added according to the manufacturer’s instructions, and samples were incubated for 15 min in the dark at room temperature. Stained cells were analyzed immediately by flow cytometry, with at least 10,000 events collected per sample. Annexin V^+^/PI⁻ cells were considered early apoptotic, while Annexin V⁺/PI⁺ cells were classified as late apoptotic.

### RNA sequencing and analysis

Bulk RNA sequencing was performed on BMDMs from the Control, MD, and MD + FGF4 groups by OE Biotech Co., Ltd. (Shanghai, China). Clean reads were obtained after quality control and aligned to the mouse reference genome (GRCm39). Gene expression levels were quantified, and differentially expressed genes were identified using DESeq2 (adjusted q < 0.05, |log2FC| > 1). GO and KEGG pathway enrichment analyses were conducted based on the identified gene set.

### RNA isolation and quantitative real-time PCR

Total RNA was extracted from BMDMs using the EZ-press RNA Purification Kit (EZBioscience, B0004DP) following the supplier’s column-based instructions. Spectrophotometry measured RNA amount and purity. To create complementary DNA (cDNA), 1 µg of total RNA was reverse-transcribed using the iScript cDNA Synthesis Kit (Bio-Rad) at recommended temperatures (25 °C for 5 min, 46 °C for 20 min, and 95 °C for 1 min). On a real-time PCR apparatus, SYBR Green Master Mix (Thermo Fisher Scientific, 2741382) was used for qPCR. Each 10 µL reaction mixture contained diluted cDNA template, gene-specific primers, SYBR Green solution, and nuclease-free water. The amplification process involved initial incubation at 50 °C for 2 min and 95 °C for 2 min, followed by 40 cycles of denaturation at 95 °C for 15 s, primer annealing at optimum temperature (< 60 °C) for 15 s, and extension at 72 °C for 1 min. Melt-curve analysis was conducted to verify amplification specificity. β-actin served as the internal reference gene, and relative transcript levels were calculated using the 2^^^ − ΔΔCt method. Primer sequences are provided in Supplementary Table S1.

### Lysosomal membrane permeabilization (LMP) assay

Lysosomal membrane integrity was assessed using the BBcellProbe AO LMP/Integrity Assay Kit (Beibo, BB-48142). AO dye was diluted 1:10 with the dye dilution buffer and then further diluted 1:20 with PBS to prepare the working solution. Subsequent to treatment, cells were rinsed once with PBS and incubated with the AO working solution at 37 °C for 30 min. Cells were subsequently washed 2 times with cold washing buffer, and lysosomal membrane permeabilization was evaluated by measuring AO red fluorescence (Ex: 555 nm, Em: 616 nm). A decrease in red fluorescence intensity indicates increased lysosomal membrane permeability.

### Lysosomal membrane protein extraction

Lysosomal membrane proteins were isolated from BMDMs using the Lysosomal Membrane Protein Extraction Kit (Beibo, BB-314523) following the manufacturer’s protocol. Briefly, 1–2 × 10⁷ cells were collected, washed with cold PBS, and incubated with reagent A on ice, followed by homogenization with a Dounce homogenizer. The homogenate was centrifuged successively at 4 ℃ at 1000 × g (to obtain the supernatant), 4000 × g (to obtain the supernatant), 20,000 × g, and the precipitate was collected to obtain the lysosomal particles. The pellet was washed with reagent B, centrifuged again, and then lysed with extraction buffer C at 2–8 °C. After centrifugation at 12,000 × g for 5 min, the supernatant was incubated at 37 °C for 10 min and centrifuged at 1000 × g to separate the lysosomal membrane protein fraction. The lower phase was dissolved in reagent E and used for subsequent analyses.

### Assessment of locomotor function after spinal cord injury

Hindlimb motor recovery after spinal cord injury was evaluated using the Basso Mouse Scale (BMS) at 1, 3, 7, 10, 14, 21, 28, 35, and 42 days post-injury, with scoring performed by three blinded investigators. At 42 days, gait patterns were assessed using a footprint test in which mice walked along an 8 × 50 cm paper-covered walkway with hind paws stained red, and the resulting footprints were scanned for analysis. Hindlimb kinematics were further examined at 42 days by recording high-speed videos (120 fps) and tracking five anatomical points using DeepLabCutGUI 2.3.5; stick-like reconstructions and joint angle trajectories were generated in MATLAB R2023b, and gait parameters such as foot placement error and standing height were quantified.

### Motor evoked potential (MEP) recording

Motor evoked potentials (MEPs) were recorded to assess spinal conduction after injury. Mice were anesthetized, and transcranial stimulation was applied using needle electrodes placed over the motor cortex with constant-current pulses (0.5 mA, 0.1 ms). Recording electrodes were inserted into the gastrocnemius muscle, with a reference electrode placed nearby. For each animal, 3–5 stable responses were collected and averaged. MEP amplitude were analyzed by investigators blinded to group allocation.

### Statistical analysis

Data were analyzed using GraphPad Prism 8.4 and are presented as mean ± SD. Normality was assessed using the Shapiro–Wilk test, and variance homogeneity was assessed using the Brown–Forsythe test. For normally distributed data, two-tailed Student’s t-test was used for comparisons between two groups, and one-way or two-way ANOVA followed by Tukey’s post hoc test was used for multiple-group comparisons. When assumptions for parametric tests were not met, appropriate non-parametric tests were applied. Statistical significance was defined as *P* < 0.05. In all bar charts, each dot represents an individual animal (in vivo) or an independent culture (in vitro). Animals were randomized to groups, and outcome assessments were performed blinded.

## Results

### Endogenous FGF4 exhibits early transient upregulation and is associated with macrophage-mediated myelin debris clearance after SCI

**Fig. 1:**
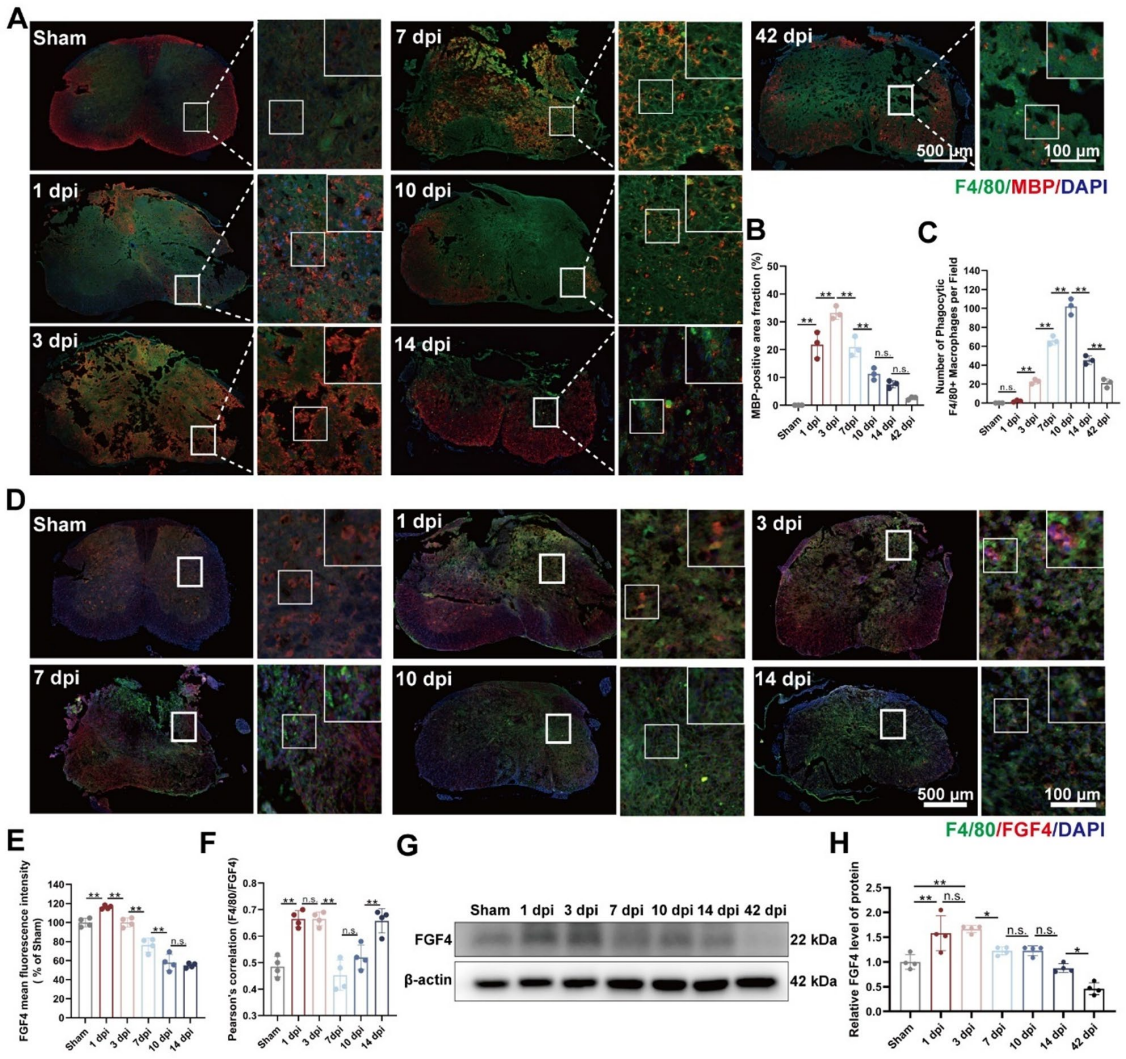
Time-dependent evolution of myelin debris phagocytosis by macrophages and endogenous FGF4 expression following spinal cord injury. **A** Representative immunofluorescence images of transverse spinal cord sections at the lesion epicenter from sham-operated mice and at 1, 3, 7, 10, 14, and 42 days post-injury (dpi). Sections were stained for macrophages (F4/80, green), myelin debris (MBP; red), and nuclei (DAPI, blue). Scale bars: 500 μm and 100 μm (magnified insets). **B** Quantitative analysis of MBP-positive area fraction (%) (*n* = 3 animals). **C** Quantitative analysis of the number of phagocytic F4/80⁺ macrophages per field (*n* = 3 animals). **D** Representative immunofluorescence images showing the localization of macrophages (F4/80, green) and FGF4 (red) in spinal cord sections from sham and injured mice at 1, 3, 7, 10, and 14 dpi. Nuclei are stained with DAPI (blue). Scale bars: 500 μm and 100 μm (magnified insets). **E** Quantitative analysis of the mean fluorescence intensity of FGF4 (*n* = 4 animals). **F** Pearson’s correlation analysis between the fluorescence signals of F4/80 and FGF4 across the examined time points (*n* = 4 animals). **G** Representative Western blot images of FGF4 protein expression in spinal cord tissue lysates from sham and injured mice at the indicated time points. β-actin served as the loading control. **H** Densitometric quantification of relative FGF4 protein levels normalized to β-actin (*n* = 4 animals). *p < 0.05, **p < 0.01, n.s. = not significant

To characterize the temporal dynamics of myelin debris clearance after spinal cord injury, we first examined the distribution of myelin debris and the phagocytic response of macrophages across multiple time points. Immunofluorescence staining showed a rapid accumulation of MBP⁺ myelin debris at the lesion site after SCI, with the overall MBP⁺ burden (MBP-positive area fraction) peaking at 3 dpi and progressively declining thereafter (Fig. 1A and B). In parallel, the number of phagocytic F4/80⁺ macrophages per field increased during the subacute phase and reached a maximum at 10 dpi, followed by a gradual decrease at later stages (Fig. 1A and C). Notably, despite the reduction in MBP⁺ burden, residual MBP⁺ signals and phagocytic macrophages remained detectable at 42 dpi, indicating that degenerating myelin debris is not completely cleared even at late stages (Fig. 1A–C).

We subsequently evaluated the endogenous expression of FGF4 following injury. At 1 dpi, FGF4 immunoreactivity rose sharply, then slowly fell over the next few days (Fig. 1D and E). Pearson’s correlation analysis revealed that at 1 and 3 dpi, the co-localization phenomenon of F4/80 and FGF4 was enhanced, indicating that FGF4 was enriched in the macrophage aggregation area at an early stage. Notably, the correlation coefficient decreased sharply at the beginning of enhanced macrophage phagocytosis at 7 dpi, followed by a slow rebound (Fig. 1F).

In line with the immunofluorescence results, Western blot analysis showed that FGF4 protein levels were much higher at 1 and 3 dpi than in the sham group. They then stayed lower as the injury got worse (Fig. 1G and H). These findings demonstrate that while FGF4 levels are temporarily heightened immediately following spinal cord injury (SCI), they swiftly decline thereafter. This indicates inadequate endogenous FGF4 supplementation during phases of vigorous macrophage-mediated myelin debris clearance.

### FGF4 enhances myelin debris phagocytosis in BMDMs and attenuates neuronal apoptosis induced by macrophage-conditioned medium

**Fig. 2:**
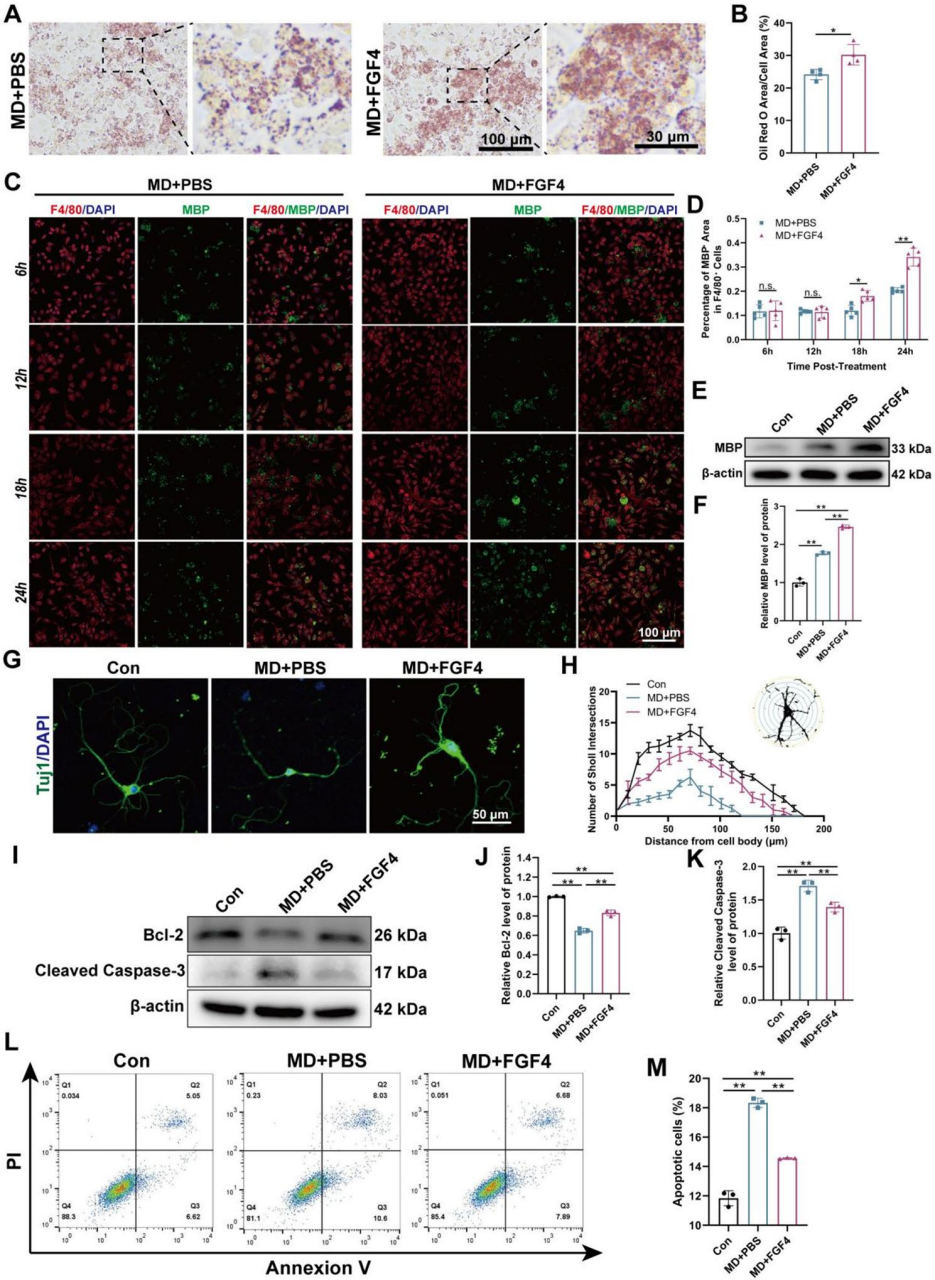
FGF4 augments myelin debris phagocytosis in BMDMs and reduces neuronal apoptosis. **A** Representative images of Oil Red O staining in BMDMs treated with myelin debris (MD) and either phosphate-buffered saline (PBS, MD + PBS group) or fibroblast growth factor 4 (FGF4, MD+FGF4 group). Scale bars: 100 μm and 30 μm (magnified inset). **B** Quantitative analysis of the Oil Red O-positive area normalized to the total cell area (*n* = 4 wells). **C** Representative immunofluorescence images of BMDMs in the MD + PBS and MD+FGF4 groups at 6, 12, 18, and 24 h post-stimulation. Cells were stained for the macrophage marker F4/80 (red), myelin basic protein (MBP, green), and nuclei (DAPI, blue). Scale bar: 100 μm. **D** Quantitative analysis of the percentage of MBP^+^ area within F4/80^+^ cells (*n* = 5 wells). **E** Representative Western blot images showing the protein levels of MBP in BMDM lysates from the Con, MD + PBS, and MD+FGF4 groups. β-actin was used as a loading control. **F** Densitometric quantification of relative MBP protein levels normalized to β-actin (*n* = 3 wells). **G** Representative immunofluorescence images of primary cortical neurons treated with conditioned medium from different groups of BMDMs, stained with the neuronal marker Tuj1 (green) and the nuclear marker DAPI (blue). Scale bar: 50 μm. **H** Sholl analysis of neuronal branching complexity. The number of neurite intersections with concentric circles at increasing distances from the soma is plotted for each group (*n* = 3 wells). **I** Representative Western blot images showing the protein levels of the anti-apoptotic protein Bcl-2 and the apoptotic marker Cleaved Caspase-3 in primary cortical neurons treated with conditioned medium from different groups of BMDMs. β-actin was used as a loading control. **J** Densitometric quantification of relative Bcl-2 protein levels normalized to β-actin (*n* = 3 wells). **K** ​Densitometric quantification of relative Cleaved Caspase-3 protein levels normalized to β-actin (*n* = 3 wells). **L** Representative flow cytometry plots of primary cortical neurons stained with Annexin V and propidium iodide (PI) after treatment with conditioned media collected from different groups of BMDMs to assess apoptosis. **M** Quantitative analysis of the total apoptotic cell rate (sum of early and late apoptotic cells, *n* = 3 wells). *p < 0.05, **p < 0.01, n.s. = not significant

To ascertain if FGF4 directly influences the phagocytic capacity of macrophages, Oil Red O staining was performed first to assess lipid droplet accumulation subsequent to myelin debris uptake. BMDMs treated with FGF4 showed a significant rise in Oil Red O–positive lipid droplets compared to the MD + PBS group, indicating enhanced degradation of phagocytosed myelin debris (Fig. 2A and B). Immunofluorescence analysis consistently showed that FGF4 greatly increased the internalization of myelin debris by macrophages. The disparity in MBP^+^ signal among F4/80^+^ cells began to show a difference at 18 h and became more pronounced at 24 h post-stimulation (Fig. 2C and D). Using Dil-labeled myelin debris, short-term uptake analysis at 37 °C for 60 min showed a significantly higher intracellular Dil-myelin area fraction in the MD+FGF4 group compared with MD + PBS (Supplementary Fig. S2), indicating that FGF4 enhances the early internalization of myelin debris by BMDMs. Western blot analysis also showed that BMDMs from the MD+FGF4 group had higher levels of MBP than those from the MD + PBS group (Fig. 2E and F). This supports the idea that FGF4 enhances macrophage-mediated myelin debris phagocytosis.

Next, we examined whether the burden of residual myelin debris in macrophage-conditioned medium influenced neuronal morphology and survival. When primary cortical neurons were grown in conditioned medium from MD-treated BMDMs, their neurite length and branching complexity were greatly reduced, as shown by Tuj1 immunostaining and Sholl analysis (Fig. 2G and H). Western blot results demonstrated a corresponding decrease in the anti-apoptotic protein Bcl-2 and an increase in Cleaved Caspase-3 (Fig. 2I–K), further indicating enhanced neuronal stress and apoptosis. Flow cytometry confirmed a significant rise in the proportion of apoptotic neurons in this group (Fig. 2L and M). Significantly, conditioned medium from BMDMs pretreated with FGF4 before exposure to myelin debris maintained neuronal morphology, enhanced neurite complexity, and decreased neuronal apoptosis in all assays. These results show that FGF4 not only helps macrophages eat up myelin debris, but it also attenuates the secondary neurotoxicity that happens when debris isn’t cleared up quickly enough, which protects the structure and health of neurons.

### FGF4 enhances myelin debris clearance and reduces lipid accumulation after SCI, accompanied by a shift toward anti-inflammatory macrophage polarization

**Fig. 3:**
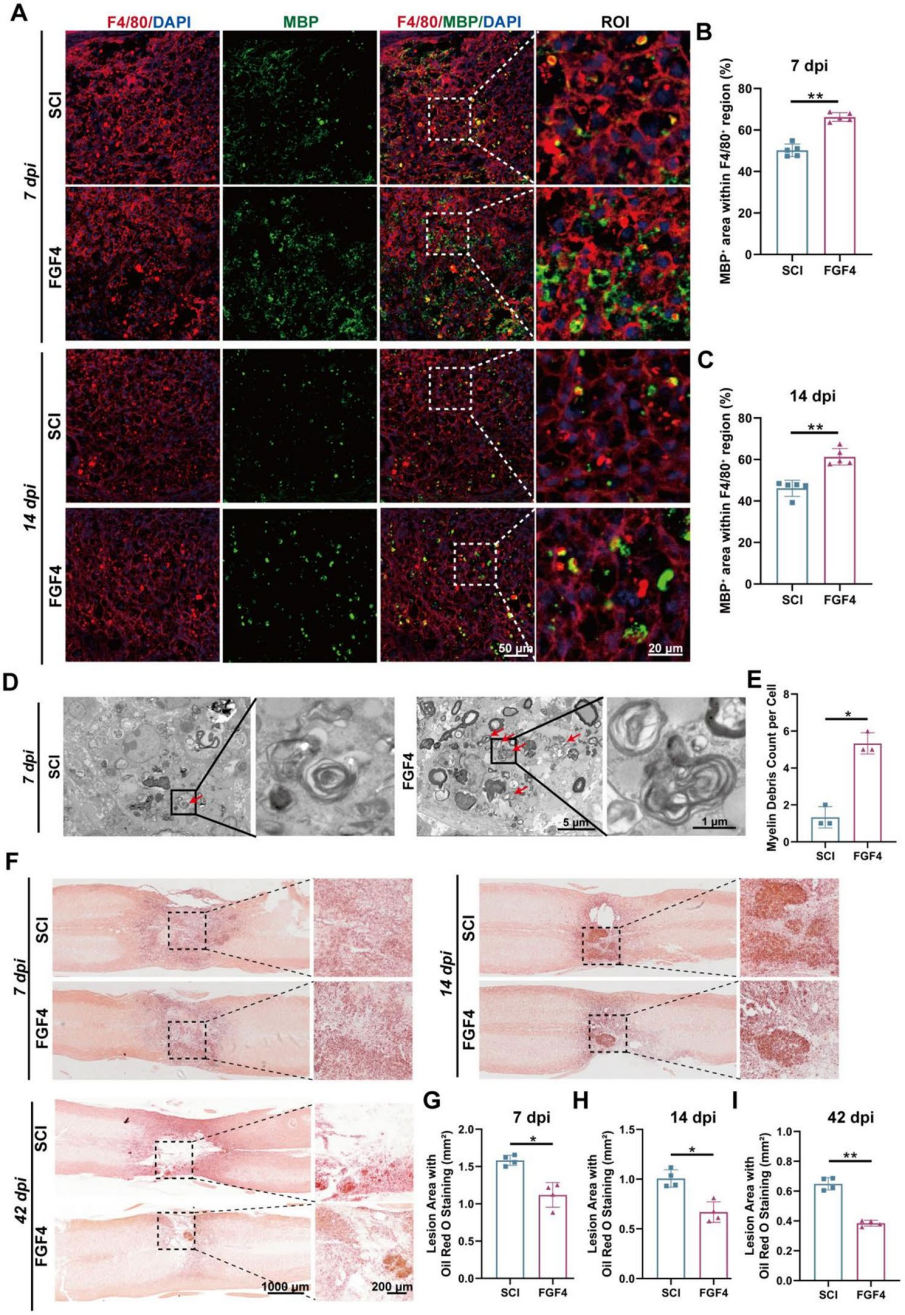
FGF4 improves in vivo myelin debris resolution and attenuates lipid accumulation in phagocytes after SCI. **A** Representative immunofluorescence images of spinal cord sections at 7 and 14 dpi, comparing the SCI group to the FGF4 group. Sections were stained for macrophages (F4/80, red), myelin basic protein (MBP, green), and nuclei (DAPI, blue). The boxed regions magnified in the corresponding right panels. Scale bars: 50 μm, 20 μm (magnified). **B** Quantitative analysis of the MBP^+^ area within F4/80^+^ regions at 7 dpi (*n* = 5 animals). **C** Quantitative analysis of the MBP^+^ area within F4/80^+^ regions at 14 dpi (*n* = 5 animals). **D** Transmission electron microscopy (TEM) images of macrophages in the spinal cord lesion area at 7 dpi. Representative images from the SCI group and the FGF4 group are shown. The boxed regions are magnified on the right, revealing degraded myelin debris (indicated by red arrows). Scale bars: 5 μm, 1 μm (magnified). **E** Quantitative analysis of the number of degraded myelin debris per macrophage from the TEM images (*n* = 3 animals). **F** Representative images of Oil Red O staining in spinal cord sections at 7, 14, and 42 dpi, comparing the SCI group and the FGF4 group. Lipid deposits are stained red. Higher-magnification views of the boxed regions displayed on the right. Scale bars: 1000 μm, 200 μm (magnified). **G-I** Quantitative analysis of the Oil Red O-positive lesion area (mm²) at 7 **(G)**, 14 **(H)**, and 42 **(I)** dpi for the indicated groups (*n* = 4 animals). *p < 0.05, **p < 0.01

To assess the effect of FGF4 on the phagocytic processing of myelin debris in vivo, we analyzed the distribution of MBP^+^ debris within F4/80^+^ macrophages at 7 and 14 dpi. Immunofluorescence staining demonstrated that FGF4 treatment significantly elevated the quantity of MBP^+^ debris internalized by macrophages at 7 dpi, indicating enhanced phagocytic activity relative to the SCI group (Fig. 3A and B). Even though the total amount of debris decreased by 14 dpi, FGF4-treated mice still had more phagocytosed MBP^+^ debris than SCI group. This means that FGF4 keeps macrophages engaged in phagocytosis during tissue remodeling (Fig. 3A and C). Besides, to distinguish infiltrating monocyte-derived macrophages (MoDMs) from resident microglia in vivo, we co-stained spinal cord sections with Ly6C or TMEM119 together with MBP. FGF4 treatment significantly increased the MBP⁺ signal within the Ly6C⁺ region at the lesion site (Supplementary Fig. S3A and B), whereas the MBP⁺ signal within the TMEM119⁺ region was not significantly changed (Supplementary Fig. S3C and D). These data suggest that FGF4-enhanced myelin debris handling is primarily attributable to infiltrating Ly6C⁺ MoDMs rather than resident microglia. Ultrastructural analysis further supported these findings. Transmission electron microscopy (TEM) images revealed a significantly greater number of degraded myelin debris within macrophages in the FGF4 group compared with the SCI group (Fig. 3D and E).

Next, we looked at how lipids built up at the site of the lesion because myelin debris degradation releases a large lipid load [34]. Oil Red O staining showed that SCI mice had a lot of lipids in the injured area at 7, 14, and 42 dpi. In contrast, FGF4-treated mice had less lipids in the injured area at all time points (Fig. 3F–I). These results show that FGF4 does not make lipid overload worse; instead, it helps the injured spinal cord process and clear lipids more quickly.

Because there is a certain correlation between the polarization types of macrophages and their ability to process myelin debris [35], we looked into whether FGF4 changes the polarization of macrophages. SCI caused a big rise in the pro-inflammatory marker CD86, but the reparative marker CD206 stayed low. Immunofluorescence and Western blot analysis showed that FGF4 treatment greatly lowered CD86 expression and raised CD206 expression at both 7 and 14 dpi (Supplementary Fig. S4). The transformation of macrophages into an anti-inflammatory, pro-repair phenotype corresponds with the enhancements in the removal of myelin debris and lipid management. This means that FGF4 helps the area around the lesion process myelin debris by making macrophages more likely to repair themselves.

These results show that FGF4 speeds up the removal of myelin debris, lowers the buildup of harmful lipids, and at the same time, pushes macrophages to become reparative, which all work together to make the environment better for tissue repair after SCI.

### FGF4 enhances lipid efflux, alleviates inflammasome-driven pyroptosis, and restores lysosomal function after SCI

**Fig. 4:**
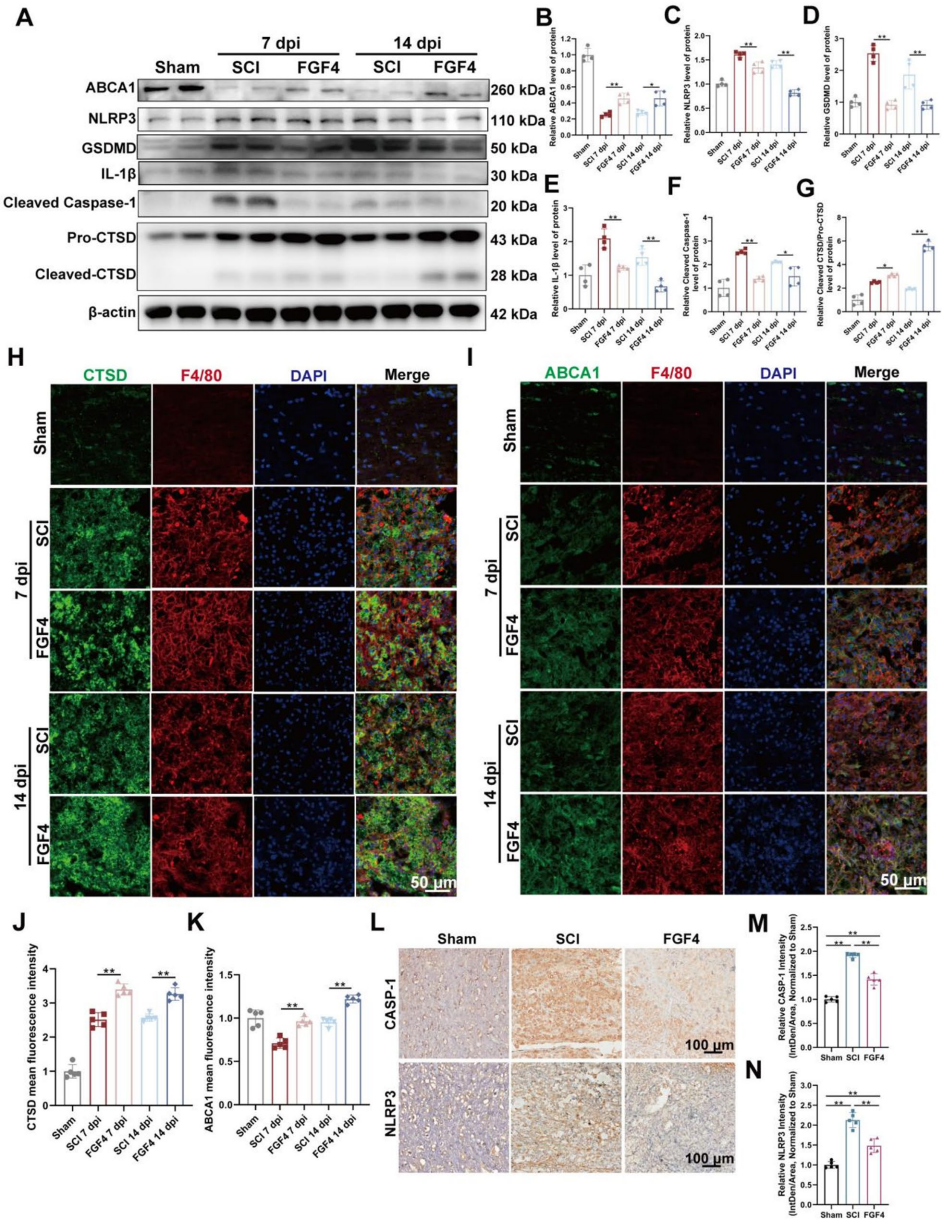
FGF4 enhances cholesterol efflux, suppresses inflammasome activation and pyroptosis, and promotes the repair of lysosomal function after SCI. **A** Representative Western blot images of proteins related to cholesterol efflux (ABCA1), inflammasome activation (NLRP3, Cleaved Caspase-1, IL-1β), pyroptosis (GSDMD), and lysosomal function (Pro-CTSD, Cleaved-CTSD) in spinal cord tissue lysates from sham-operated mice and mice subjected to SCI with or without FGF4 treatment, assessed at 7 and 14 dpi. β-actin was used as a loading control. **B–G** Quantification of relative protein levels normalized to β-actin: ABCA1 **(B)**, NLRP3 **(C)**, GSDMD **(D)**, IL-1β **(E)**, Cleaved Caspase-1 **(F)**, The ratio of Cleaved-CTSD to Pro-CTSD **(G)** (*n* = 4 animals). **H** Representative immunofluorescence staining for CTSD (green) and the macrophage marker F4/80 (red) in spinal cord sections. Nuclei are stained with DAPI (blue). Scale bar: 50 μm. **I** Representative immunofluorescence staining for ABCA1 (green) and F4/80 (red) in spinal cord sections. Nuclei are stained with DAPI (blue). Scale bar: 50 μm. **J** Quantification of the mean fluorescence intensity of CTSD (*n* = 5 animals). **K** Quantification of the mean fluorescence intensity of ABCA1 (*n* = 5 animals). **L** Representative immunohistochemical staining of CASP-1 and NLRP3 in spinal cord tissues from the indicated groups at 7 dpi. Scale bar: 100 μm. **M** Quantification of relative CASP-1 intensity (Integrated Density/Area, normalized to Sham, *n* = 5 animals). **N** Quantification of relative NLRP3 intensity (Integrated Density/Area, normalized to Sham, *n* = 5 animals). *p < 0.05, **p < 0.01

To find out how FGF4 changes the microenvironment after an injury, we looked at important molecules of lipid metabolism, inflammasome activation, pyroptosis, and lysosomal function in spinal cord tissues. Western blot results showed that ABCA1, a major transporter responsible for exporting cholesterol and preventing lipid overload in phagocytes, was significantly upregulated in FGF4-treated mice at both 7 and 14 dpi compared with the SCI group (Fig. 4A and B). This increase indicates that FGF4 not only facilitates the uptake of myelin debris but also augments downstream lipid efflux, thereby mitigating secondary lipid toxicity.

On the other hand, proteins linked to inflammasome activation and pyroptosis, such as NLRP3, GSDMD, Cleaved Caspase-1, and IL-1β, were strongly increased after SCI but decreased after FGF4 treatment at both time points (Fig. 4A and C - F). Since NLRP3 inflammasome activation and GSDMD cleavage are two of the main things that cause inflammatory pyroptotic cell death [36], the fact that they are lower means that FGF4 can stop inflammatory activation and prevent cell from pyroptosis.

Since the efficient degradation process of myelin debris by phagocytes requires complete lysosomal protein hydrolysis, we further investigated CTSD (Cathepsin D), a key lysosomal protease, whose cleavage form reflects the activated state of the lysosome [37]. FGF4 significantly increased the ratio of Cleaved-CTSD/Pro-CTSD at 7 days and 14 days (Fig. 4A and G), indicating that FGF4 enhanced the activity of the lysosome. This was supported by immunofluorescence showing elevated CTSD expression within F4/80^+^ macrophages in the FGF4 group (Fig. 4H and J). Similarly, ABCA1 immunostaining confirmed increased lipid efflux capacity in macrophages after FGF4 treatment (Fig. 4I and K). Finally, immunohistochemical staining further verified the inhibitory effect of FGF4 on pyroptosis. Expression of CASP-1 and NLRP3, two critical inflammasome components responsible for IL-1β maturation and pyroptosis initiation, was substantially reduced in FGF4-treated mice compared with SCI group at 7 dpi (Fig. 4L–N).

Collectively, these findings demonstrate that FGF4 enhances macrophage lipid handling by increasing ABCA1-mediated cholesterol and lipid efflux, mitigates inflammasome activation and pyroptosis, and promotes restoration of lysosomal function—three convergent processes essential for efficient myelin debris clearance and inflammation resolution after SCI.

### FGF4 promotes long-term structural repair and improves locomotor function after SCI

To assess long-term structural outcomes, macroscopic observation and H&E staining at 42 dpi showed large lesion cavities in the SCI group, whereas FGF4-treated mice exhibited smaller cavities and better-preserved spinal cord architecture (Supplementary Fig. S5A–B and E). Luxol Fast Blue staining confirmed greater myelin retention after FGF4 treatment (Supplementary Fig. S5C and F). Bladder histology further revealed that the detrusor muscle layer was markedly thinned in SCI mice but significantly thicker in the FGF4 group, indicating partial restoration of bladder function (Supplementary Fig. S5D and G). Immunofluorescence staining demonstrated increased NF200^+^ axons and NeuN^+^ neuronal preservation, along with reduced GFAP^+^ astrogliosis, in FGF4-treated mice (Supplementary Fig. S5H–L). Synaptic and neurotransmitter markers (Synapsin, MAP2, 5-HT, TH) were also more abundant in the FGF4 group, suggesting improved circuit reconstruction (Supplementary Fig. S5M). Western blot results confirmed elevated NF200 and MAP2 protein expression (Supplementary Fig. S5N–P).

**Fig. 5:**
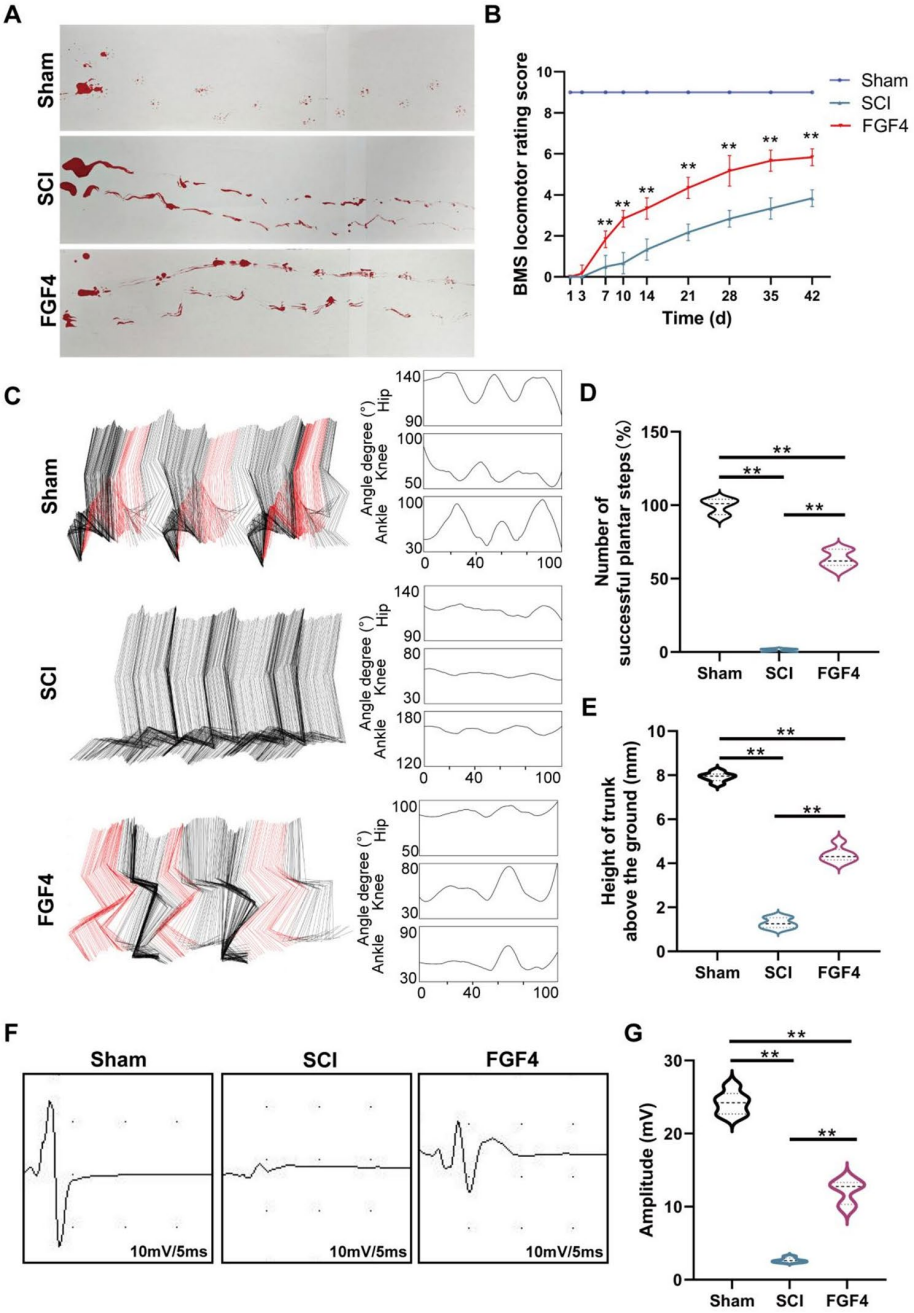
FGF4 treatment improves locomotor functional recovery following SCI. **A** Representative footprint images of hindlimb steps from the Sham, SCI, and FGF4 groups at 42 dpi. **B** Basso Mouse Scale (BMS) scores for hindlimb motor function assessed at 1, 3, 7, 10, 14, 21, 28, 35, and 42 dpi in the Sham, SCI, and FGF4 groups (*n* = 6 animals). **C** Representative kinematic analysis of hindlimb locomotion. Color-coded simulate limb movement, and joint angle curves for the hip, knee, and ankle joints are plotted. **D** Quantitative analysis of the number of successful plantar steps (*n* = 6 animals). **E** Quantitative analysis of the height of the trunk above the ground (mm, *n* = 6 animals). **F** Representative motor evoked potential (MEP) traces recorded from the Sham, SCI, and FGF4 groups at 42 dpi. **G** Quantification of MEP amplitude (mV, *n* = 6 animals). **p < 0.01

In line with these histological advancements, FGF4 also facilitated locomotor recovery. Footprint analysis indicated disorganized stepping and inadequate plantar placement in SCI mice, whereas FGF4-treated mice exhibited more continuous footprints (Fig. 5A). During the recovery period, the BMS scores showed that the FGF4 group had much better hindlimb function (Fig. 5B). Kinematic stick-figure analysis showed that SCI mice had almost no joint flexion. In contrast, FGF4-treated animals had clearer movements at the hips, knees, and ankles, with larger joint angle amplitudes (Fig. 5C). Quantitative analysis validated increased successful plantar steps (Fig. 5D) and elevated trunk height during locomotion (Fig. 5E) subsequent to FGF4 treatment. Electrophysiological evaluation revealed markedly greater MEP amplitudes in the FGF4 group, signifying enhanced conduction in descending motor pathways (Fig. 5F–G).

These findings collectively demonstrate that FGF4 enhances long-term tissue repair following spinal cord injury by diminishing lesion cavities, safeguarding myelin and neurons, and fostering axonal and synaptic integrity, thereby facilitating superior locomotor recovery.

### FGFR1 inhibition abrogates FGF4-mediated PI3K/AKT activation and phagocytic enhancement in macrophages

**Fig. 6:**
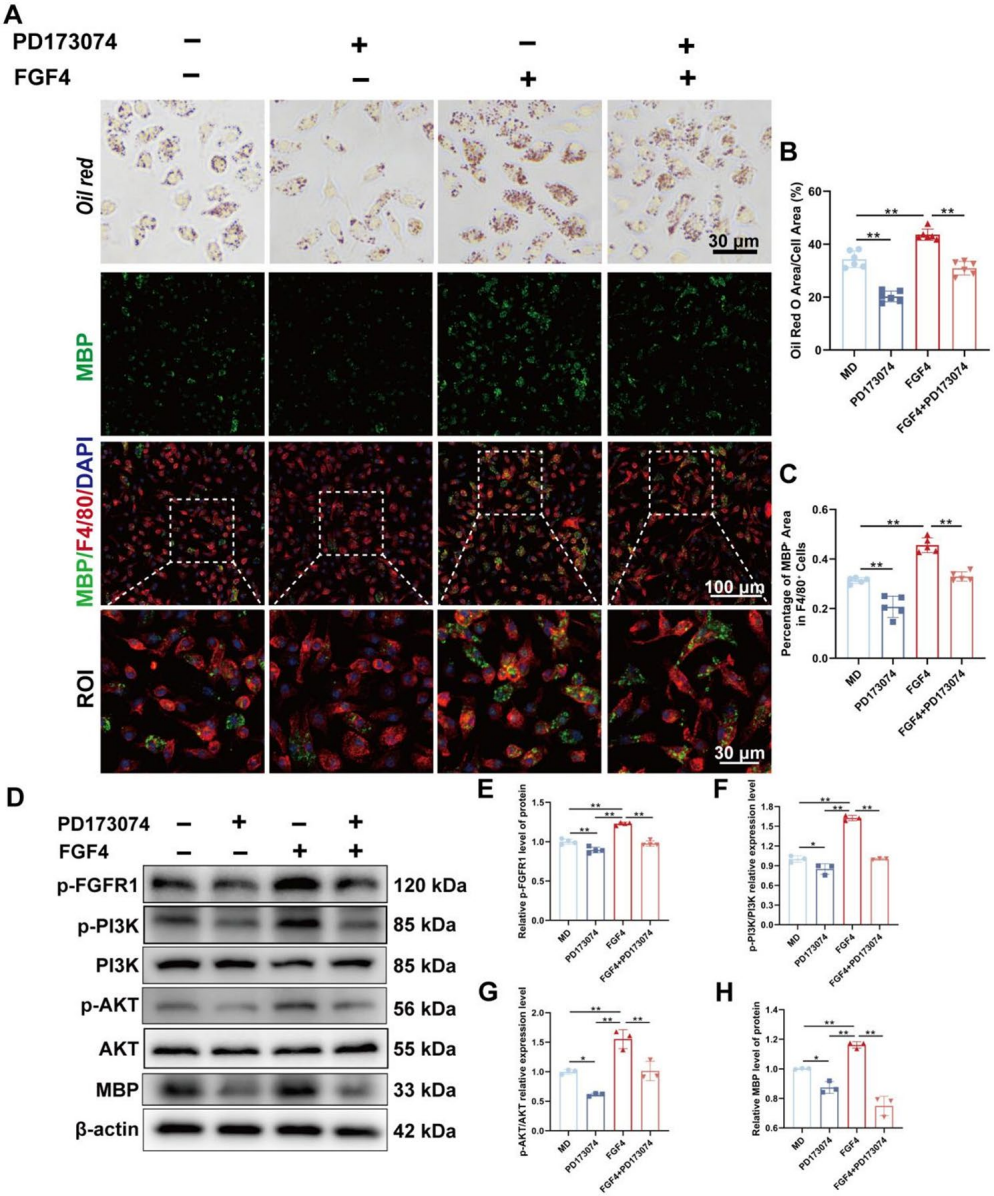
FGFR1 inhibition abrogates FGF4-mediated PI3K/AKT activation and phagocytic enhancement in macrophages. **A** Representative Oil Red O staining of BMDMs under PD173074 (FGFR1 inhibitor) treatment. Scale bar: 30 μm. Representative immunofluorescence images stained for myelin debris (MBP, green), macrophages (F4/80, red), and nuclei (DAPI, blue). Boxed regions are magnified below. Scale bars: 100 μm, 30 μm (magnified). **B** Quantitative analysis of the Oil Red O-positive area expressed as a percentage (%) of the total cell area (*n* = 6 wells). **C** Quantitative analysis of the percentage of MBP^+^ area within F4/80^+^ cells (*n* = 5 wells). **D** Representative Western blot images of phosphorylated FGFR1 (p-FGFR1), phosphorylated PI3K (p-PI3K), total PI3K, phosphorylated AKT (p-AKT), total AKT, MBP, and β-actin in BMDM lysates under PD173074 treatment. **E–H** Densitometric quantification of relative protein levels: p-FGFR1 **(E)** (*n* = 4 wells), p-PI3K/PI3K ratio **(F)** (*n* = 3 wells), p-AKT/AKT ratio **(G)** (*n* = 3 wells), MBP **(H)** (*n* = 3 wells). All data normalized to β-actin. *p < 0.05, **p < 0.01

We used the FGFR1 blocker PD173074 on BMDMs to find out if FGFR1 signaling is needed for FGF4’s pro-phagocytic effects. Oil Red O staining showed that after taking in myelin debris, FGF4 caused a lot more lipid droplets to build up. PD173074 attenuated lipid droplet formation. In addition, the combination of PD173074 and FGF4 reduced lipid droplet formation compared with the FGF4 treatment group, implying that the phagocytosis mediated by FGF4 was hindered (Fig. 6A and B). Immunofluorescence analysis also showed that FGF4 increased the internalization of MBP^+^ myelin debris in F4/80^+^ macrophages, while PD173074 treatment greatly decreased MBP uptake, reversing the effect of FGF4 (Fig. 6A and C).

Western blot analysis showed that FGF4 strongly activated the FGFR1–PI3K/AKT signaling pathway by showing higher levels of p-FGFR1, higher p-PI3K/PI3K ratios, and higher p-AKT/AKT ratios. Along with these molecular changes, the levels of MBP protein went up, which is in line with increased phagocytic activity (Fig. 6D - H). Importantly, PD173074 treatment markedly reduced p-FGFR1 and downstream PI3K/AKT phosphorylation, and also decreased MBP accumulation, confirming that FGF4 requires FGFR1 activation to promote PI3K/AKT signaling and phagocytic enhancement. Consistent with in vivo target engagement, at 7 dpi immunofluorescence co-staining showed increased p-FGFR1 and p-AKT signals within F4/80⁺ lesion-associated macrophages after FGF4 administration (Supplementary Fig. S6A - D). Western blotting of injured spinal cord tissue at 7 dpi further confirmed activation of the FGFR1–PI3K/AKT axis (Supplementary Fig. S6E - H).

These results demonstrate that inhibition of FGFR1 effectively blocks the ability of FGF4 to activate PI3K/AKT signaling and to augment macrophage phagocytosis of myelin debris, indicating that FGFR1 is essential for FGF4-mediated macrophage functional enhancement.

### FGF4 reprograms macrophage receptor and lipid-handling gene expression and identifies Clec10a as a major downstream effector

**Fig. 7:**
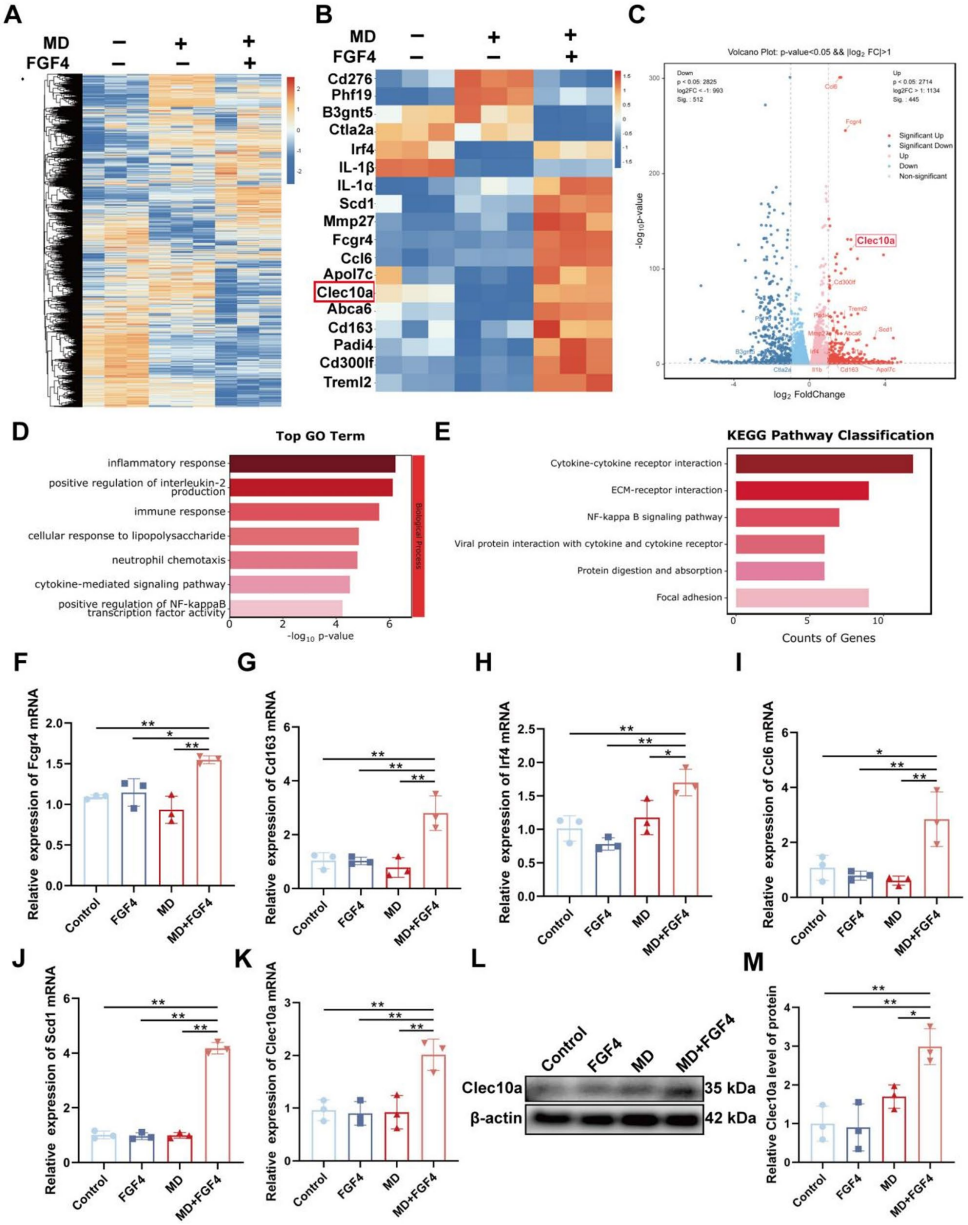
Transcriptomic profiling reveals that FGF4 reshapes macrophage receptor and lipid-handling gene expression and identifies Clec10a as a key downstream effector. **A** Summary of differentially expressed genes (DEGs) identified by RNA-seq analysis among the Control, MD, and MD+FGF4 groups. BMDMs were pretreated with FGF4 for 2 h followed by exposure to MD for 24 h. **B** Heatmap displaying the expression patterns of genes related to phagocytosis across the indicated groups. **C** Volcano plot illustrating the significance and magnitude of gene expression changes for phagocytosis-related genes between the MD+FGF4 and MD groups. **D-E** Gene Ontology (GO) biological process enrichment analysis **(D)** and Kyoto Encyclopedia of Genes and Genomes (KEGG) pathway enrichment analysis **(E)** of the phagocytosis-related DEGs. **F–K** Validation of RNA-seq data by quantitative RT-PCR (qRT-PCR). Relative mRNA expression levels of Fcgr4 **(F)**, Cd163 **(G)**, Irf4 **(H)**, Ccl6 **(I)**, Scd1 **(J)**, and Clec10a **(K)** in BMDMs from the Control, FGF4, MD, and MD+FGF4 groups. Data were normalized to β-actin and presented as relative to the Control group (*n* = 3 wells). **L** Representative Western blot images showing Clec10a protein expression in BMDM lysates. β-actin was used as a loading control. **M** Densitometric quantification of relative Clec10a protein levels normalized to β-actin (*n* = 3 wells). *p < 0.05, **p < 0.01

To investigate how FGF4 modulates macrophage gene expression during myelin debris processing, RNA-seq was performed on BMDMs exposed to MD with or without FGF4 pretreatment. Global transcriptomic analysis showed that MD stimulation induced a strong inflammatory signature, whereas FGF4 substantially reshaped this profile (Fig. 7A). A focused heatmap of receptor- and lipid-related genes revealed that several genes previously associated with macrophage activation, receptor engagement, and lipid handling—such as Fcgr4, Cd163, Irf4, Ccl6, Scd1, and particularly Clec10a—were markedly upregulated in the MD+FGF4 group compared with MD alone (Fig. 7B). Volcano plot analysis highlighted Clec10a as a prominently upregulated gene in response to FGF4 treatment (Fig. 7C).

GO enrichment analysis revealed that the differentially expressed genes were primarily grouped into inflammatory and cytokine-mediated pathways, while KEGG enrichment highlighted cytokine–receptor interactions, ECM–receptor interactions, and focal adhesion pathways (Fig. 7D and E). Although these unbiased enrichment analyses emphasize broad immune signaling rather than the specific phagocytosis pathway, many of the individual genes enhanced by FGF4—such as Fcgr4, Cd163, Irf4, Ccl6, Scd1, and Clec10a—have been functionally implicated in receptor-mediated uptake and lipid metabolic processing. These results suggest that FGF4 shifts macrophages toward a transcriptional state more permissive for debris engagement and lipid handling.

qRT-PCR validation confirmed the RNA-seq findings, showing significant upregulation of Fcgr4, Cd163, Irf4, Ccl6, Scd1, and Clec10a in the MD+FGF4 group (Fig. 7F–K). Among these, Clec10a exhibited pronounced increase at the mRNA and protein levels (Fig. 7L–M). Together, these results indicate that FGF4 enhances the expression of macrophage receptors and lipid-processing genes and highlight Clec10a as a key downstream effector that may contribute to FGF4-mediated improvement in myelin debris clearance.

### Clec10a is required for FGF4-mediated enhancement of myelin debris phagocytosis and neuronal protection

**Fig. 8:**
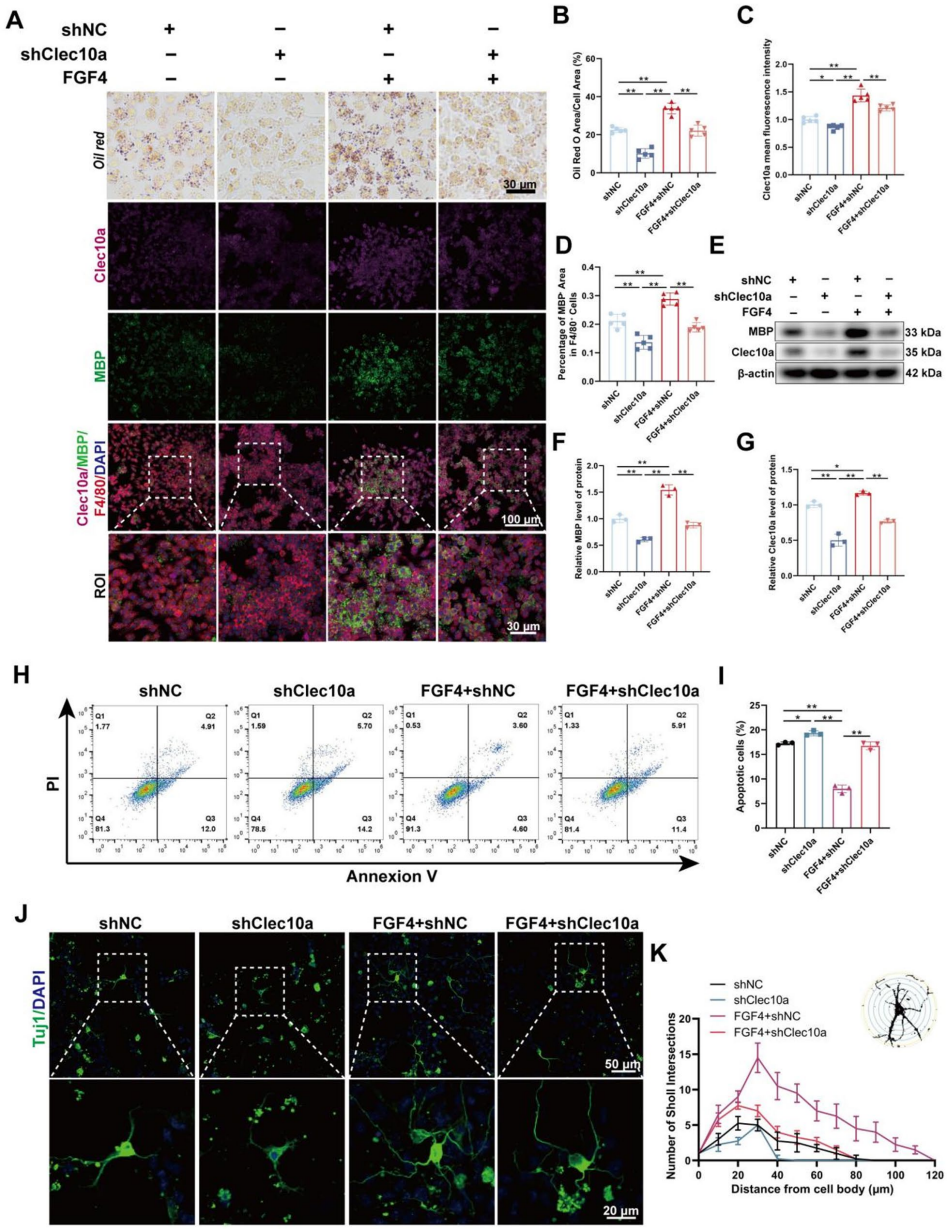
Clec10a is essential for FGF4-mediated enhancement of myelin debris phagocytosis. **A** Representative Oil Red O staining of BMDMs transfected with LV-shClec10a. Scale bar: 30 μm. Representative immunofluorescence images of BMDMs stained for Clec10a (purple), MBP (green), F4/80 (red), and DAPI (blue). Boxed regions are magnified below. Scale bars: 100 μm, 30 μm (magnified). **B** Quantitative analysis of the Oil Red O-positive area expressed as a percentage (%) of the total cell area (*n* = 5 wells). **C** Quantitative analysis of the mean fluorescence intensity of Clec10a in BMDMs (*n* = 5 wells). **D** Quantitative analysis of the percentage of MBP^+^ area within F4/80^+^ cells (*n* = 5 wells). **E** Representative Western blot images of MBP and Clec10a protein expression in BMDM lysates following LV-shClec10a transfection. β-actin was used as a loading control. **F** Densitometric quantification of relative MBP protein levels normalized to β-actin (*n* = 3 wells). **G** Densitometric quantification of relative Clec10a protein levels normalized to β-actin (*n* = 3 wells). **H** Representative flow cytometry plots of primary cortical neurons stained with Annexin V and PI after treatment with conditioned media collected from the differently treated BMDM groups. **I** Quantitative analysis of the total apoptotic cell rate (*n* = 3 wells). **J** Representative immunofluorescence images of primary cortical neurons treated with conditioned medium from different groups of BMDMs, stained with the neuronal marker Tuj1 (green) and the nuclear marker DAPI (blue). Boxed regions are magnified below. Scale bars: 50 μm, 20 μm (magnified). **K** Sholl analysis of neuronal branching complexity. The number of neurite intersections with concentric circles at increasing distances from the soma is plotted for each group (*n* = 3 wells). *p < 0.05, **p < 0.01

To determine whether Clec10a is functionally required for the pro-phagocytic effects of FGF4, BMDMs were transduced with LV-shClec10a. Oil Red O staining revealed that FGF4 markedly increased lipid droplet accumulation following myelin debris uptake in shNC-transduced cells, whereas Clec10a knockdown substantially reduced lipid accumulation and effectively blunted the enhancement induced by FGF4 (Fig. 8A and B). Immunofluorescence staining further confirmed a pronounced reduction in Clec10a expression after shRNA transduction (Fig. 8A and C), accompanied by decreased internalization of MBP^+^ myelin debris within F4/80^+^ macrophages. Notably, the FGF4-induced increase in MBP uptake was markedly attenuated in the shClec10a group (Fig. 8A and D).

Western blot analysis corroborated these findings: FGF4 enhanced MBP accumulation and increased Clec10a protein expression, whereas Clec10a knockdown significantly diminished both effects (Fig. 8E–G). These findings indicate that Clec10a is a crucial downstream effector facilitating FGF4’s enhancement of macrophage phagocytosis of myelin debris.

To evaluate the functional influence of Clec10a on neuron–macrophage interactions, conditioned medium from distinct BMDM groups was administered to primary cortical neurons. Flow cytometry analysis showed that conditioned medium from MD-treated macrophages increased neuronal apoptosis, whereas medium from FGF4-pretreated macrophages markedly reduced apoptotic rates. Importantly, Clec10a knockdown abolished this neuroprotective effect, resulting in significantly higher levels of neuronal apoptosis (Fig. 8H and I). Immunofluorescence staining and Sholl analysis further demonstrated that neurons exposed to FGF4-conditioned medium exhibited improved neurite length and branching, while medium from Clec10a-deficient macrophages severely impaired neurite complexity and eliminated the beneficial effects of FGF4 (Fig. 8J and K).

To test whether FGF4-enhanced myelin debris handling requires carbohydrate-recognition domain (CRD)–dependent ligand recognition by Clec10a, we blocked Clec10a-mediated carbohydrate recognition using N-acetylgalactosamine (GalNAc) and assessed myelin debris binding and uptake in the presence or absence of FGF4. D-GalNAc treatment did not alter myelin debris uptake in MD-treated macrophages and failed to abolish the FGF4-induced enhancement of myelin debris handling, as assessed by Oil Red O staining, immunofluorescence, and Western blotting (Supplementary Fig. S7 ).

Collectively, these findings establish that Clec10a is required for FGF4-mediated myelin debris clearance and neuroprotection, and that FGF4 enhances Clec10a- mediated intracellular processing of internalized myelin debris independently of canonical GalNAc-dependent carbohydrate recognition.

### FGF4 accelerates Clec10a-mediated phagosome maturation and restores lysosomal degradative function

**Fig. 9:**
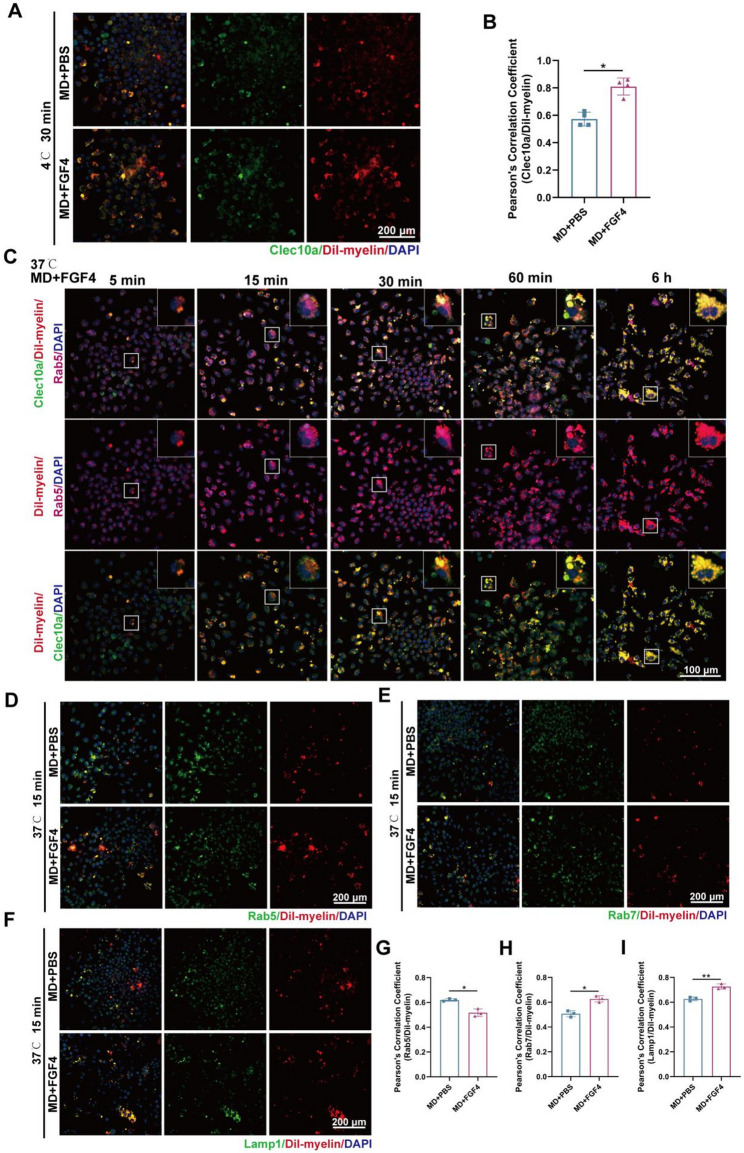
Clec10a acts intracellularly to facilitate myelin debris processing rather than through surface-level recognition. **A** Representative immunofluorescence images of BMDMs pretreated with or without FGF4 for 2 h, followed by incubation with Dil-labeled myelin debris (red) at 4 °C for 30 min to block internalization and assess surface binding. Cells were stained for Clec10a (green) and DAPI (blue). Scale bar, 200 μm. **B** Quantification of Clec10a/Dil-myelin colocalization expressed as Pearson’s correlation coefficient (*n* = 4 wells). **C** Representative immunofluorescence images of BMDMs pretreated with FGF4 for 2 h and then incubated with Dil-myelin debris at 37 °C for 5, 15, 30, 60 min, and 6 h. Cells were stained for Clec10a (green), RAB5 (purple), and DAPI (blue). Scale bar: 100 μm. **D–F** Representative images showing colocalization of Dil-myelin (red) with Rab5 (green) **(D)**, Rab7 (green) **(E)**, or Lamp1 (green) **(F)** in BMDMs pretreated with or without FGF4 and incubated with myelin debris at 37 °C for 15 min. Nuclei are stained with DAPI (blue). Scale bars: 200 μm. **G–I** Quantitative analysis of Pearson’s correlation coefficients for Rab5/Dil-myelin **(G)**, Rab7/Dil-myelin **(H)**, and Lamp1/Dil-myelin (*n* = 3 wells) **(I)**. *p < 0.05, **p < 0.01

We did binding experiments at 4 °C to stop endocytosis and see if FGF4 helps the cell process myelin debris at the surface or inside the cell. In these non-internalizing conditions, Clec10a and Dil-labeled myelin debris were primarily observed as distinct punctate structures rather than as continuous rim-like colocalization along the cell periphery (Fig. 9A). It is important to note that even though classical surface-associated binding stayed low, FGF4 pretreatment made Clec10a/Dil-myelin punctate colocalization much higher than in control cells (Fig. 9B). These results suggest that FGF4 does not primarily augment surface-level recognition of myelin debris; rather, it facilitates Clec10a-associated myelin debris management via intracellular mechanisms.

To define how FGF4 regulates the intracellular handling of myelin debris by macrophages, time-series immunofluorescence analyses were first performed. Dil-labeled myelin debris displayed progressively increased colocalization with Clec10a from 5 min to 6 h, with a clear punctate overlap already visible at 5 min, indicating rapid recruitment of Clec10a to myelin debris. Correspondingly, myelin-containing vesicles showed gradual maturation through the endosomal pathway. Dil-labeled myelin debris exhibited extensive colocalization with Rab5 as early as 15 min (Fig. 9C), indicating rapid recruitment to early endosomal compartments. This was followed by prominent colocalization with Rab7 beginning at 30 min (Supplementary Fig. S8A) and a similarly marked increase in colocalization with Lamp1^+^ lysosomes over the same time course (Supplementary Fig. S8B). These temporal patterns identified 15 min as a key transition point between early and late endosomal stages and guided its use for subsequent comparative analyses.

At 37 °C, when uptake was normal, FGF4 markedly altered intracellular trafficking. At the chosen 15-minute mark, FGF4 decreased the colocalization of Dil-myelin/Rab5 (Fig. 9D) while increasing the colocalization of Dil-myelin/Rab7 (Fig. 9E) and Dil-myelin/Lamp1 (Fig. 9F). Quantitative analyses validated that FGF4 expedites phagosome maturation, facilitating an earlier transition from Rab5^+^ early endosomes and a swifter ingress into Rab7^+^ and Lamp1^+^ late endosomal/lysosomal compartments (Fig. 9G–I). We did more lysosomal tests to see if better trafficking led to better degradative capacity. High-resolution Z-stack imaging illustrated the spatial distribution of internalized myelin debris within Rab5^+^, Rab7^+^, and Lamp1^+^ compartments, as well as CTSD, 18 h post-exposure, thereby confirming advancement through the endolysosomal pathway (Supplementary Fig. S9A - F). Functional tests of lysosomal membrane stability using AO fluorescence showed that myelin debris caused lysosomal membrane permeabilization (LMP), which was shown by a decrease in AO red fluorescence. FGF4 pretreatment significantly maintained AO fluorescence intensity, suggesting a protective effect against LMP (Supplementary Fig. S9G). Western blotting of purified lysosomal fractions showed that CTSD levels were reduced following myelin debris exposure, while FGF4 restored CTSD abundance, suggesting recovery of lysosomal proteolytic capacity. Quantification further supported that FGF4 maintains lysosomal enzyme levels in the presence of myelin debris (Supplementary Fig. S9H and I).

To define the intracellular localization of Clec10a along the endolysosomal pathway, we performed confocal Z-stack imaging and time-resolved colocalization analyses following myelin exposure. Clec10a showed intracellular association with Rab5⁺ early endosomes, Rab7⁺ late endosomes, and Lamp1⁺ lysosomes (Supplementary Fig. S10A - C), and Pearson’s correlation coefficients increased over time, becoming pronounced from 15 min after myelin exposure (Supplementary Fig. S10E - G). These data suggest dynamic recruitment of Clec10a to endolysosomal trafficking compartments during cargo processing.

Functionally, Clec10a knockdown impaired FGF4-enhanced trafficking of internalized myelin cargo. Compared with shNC, shClec10a reduced the colocalization of Dil-myelin with Rab5, Rab7, and Lamp1 under FGF4 stimulation (Supplementary Fig. S11A - F), indicating delayed progression. In parallel, Clec10a silencing blunted the FGF4-associated improvements in lysosomal integrity and proteolytic capacity, as evidenced by the AO-based lysosomal membrane permeabilization assay and decreased CTSD enrichment in purified lysosomal fractions (Supplementary Fig. S11G - I).

**Fig. 10:**
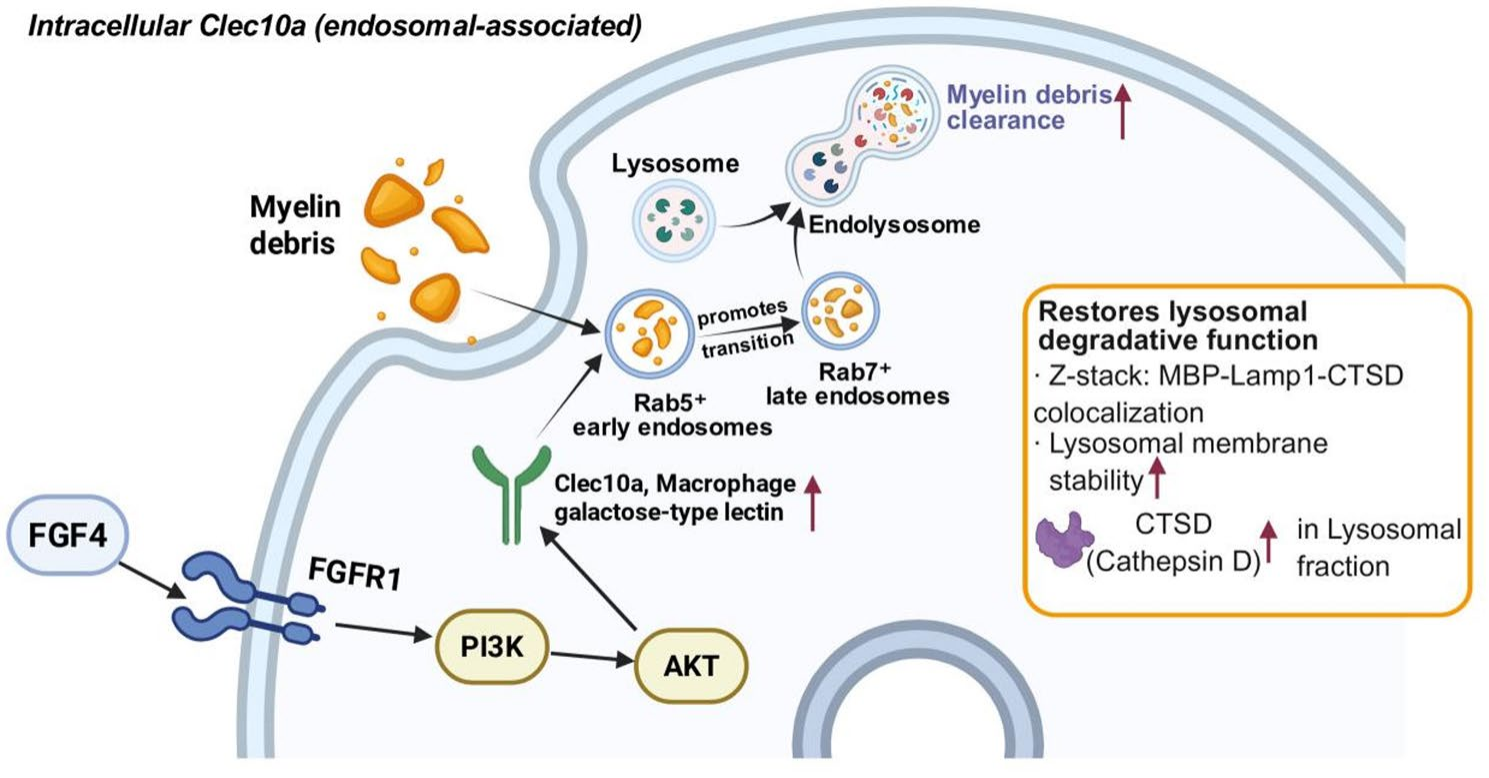
FGF4 activates FGFR1–PI3K/AKT signaling in macrophages to upregulate intracellular Clec10a, which promotes phagosome maturation and endolysosomal trafficking of internalized myelin debris from Rab5⁺ early endosomes to Rab7⁺ late endosomes and Lamp1⁺ lysosomes. This process restores lysosomal membrane stability and CTSD-dependent degradative capacity, enhances myelin debris clearance and ultimately creates a permissive microenvironment for neuronal survival and spinal cord repair. The schematic is created using BioRender

Taken together, the combined evidence demonstrates that Clec10a functions intracellularly rather than at the surface, and that FGF4 enhances myelin debris processing by accelerating phagosome maturation and stabilizing lysosomal function, thereby promoting efficient degradation of internalized myelin debris (Fig. 10).

## Discussion

The present study identifies fibroblast growth factor 4 (FGF4) as a previously unrecognized factor that regulates the phagocytosis of myelin debris by macrophages after spinal cord injury (SCI). We show that endogenous FGF4 is transiently up-regulated during the acute phase of SCI and associates spatially and temporally with infiltrating F4/80^+^ macrophages engaged in myelin debris clearance. Exogenous FGF4 further enhances myelin debris uptake by macrophages and promotes long-term tissue and functional repair in vivo. Mechanistically, FGF4 signals through the FGFR1 - PI3K/AKT axis to increase phagocytic capacity, accelerate phagosome–lysosome maturation, restore lysosomal proteolytic function. Transcriptomic and loss-of-function analyses identify the C-type lectin Clec10a as a critical downstream effector that operates in an unconventional, largely intracellular manner to facilitate myelin debris processing rather than classical carbohydrate-dependent ligand recognition. Together, these findings delineate an FGF4–FGFR1–PI3K/AKT–Clec10a pathway that couples debris clearance to metabolic and inflammatory resolution after SCI.

SCI is characterized by primary mechanical damage followed by a complex cascade of secondary events, including vascular disruption, excitotoxicity, demyelination, and chronic neuroinflammation [38]. A large body of work indicates that myelin debris is generated rapidly within hours to days after injury but persists for weeks in the lesion, where it inhibits oligodendrocyte differentiation, axonal regrowth, and remyelination [39, 40]. Some studies have shown that myelin debris phagocytosis can lead to anti-inflammatory polarization in certain situations, more and more evidence suggests that pro-inflammatory macrophage subsets are more common in lipid-rich lesions [41]. The MSR1 - NF-κB signaling pathway and other macrophage scavenger receptor-dependent pathways will be turned on when too much myelin debris is taken up. This will lead to the formation of foam cells and the continued activation of inflammation [16]. Classical M2-associated markers drop significantly during the time when foam macrophages appear, while lipid efflux regulators like ABCA1 drop after that [42]. Foam macrophages have trouble moving and eating things, and they don’t clear out apoptotic cells and neutrophils as well as they should. All of these things lead to necrosis and chronic inflammation [43, 44]. We provide evidence of abundant formation of foamy macrophages following SCI, accompanied by an M1-like phenotype. However, reduced lipid deposition with FGF4 treatment was accompanied by an increase in anti-inflammatory M2-like phenotype. These observations suggest that it is worth considering how to balance the effective clearance of myelin debris with the adverse effects produced by phagocytosis of myelin debris.

Blood-derived monocyte–macrophages get into the injured spinal cord within 1 to 3 days, reach their highest numbers around 7 to 14 days, and stay there for many weeks [45, 46]. Macrophages are essential for phagocytosing myelin debris while they also contribute to secondary damage when they assume a persistent pro-inflammatory, foam-cell-like phenotype [34]. It becomes an interesting therapeutic goal to boost macrophage activity (effective phagocytosis and timely lipid excretion) while keeping chronic macrophage inflammation to a minimum. Recent reports show that using trehalose to change macrophage autophagy or apolipoprotein A-I mimetic peptide D-4 F can help clear away myelin debris, lower the burden of foamy macrophages, and make SCI outcomes better [47, 48]. Our data show that FGF4 is similar to these methods that help macrophages get rid of myelin debris while also lowering lipid-driven toxicity.

FGFs family plays an important role in the fine regulation of glucose and lipid metabolism and response [49]. Endocrine members such as FGF21 regulate glucose and lipid metabolism by binding to FGFR1 receptor and co-receptor β-Klotho on the cell surface to stimulate the secretion of the adiponectin [50]. FGF1 can improve hepatic steatosis and reduce hepatic fat accumulation [51]. However, the effects on macrophages were not consistent, FGF12 (an autocrine growth factor) upregulates Ly6C expression in macrophages to aggravate liver fibrosis [52], while FGF18 (paracrine growth factor) induces anti-inflammatory M2 macrophages to promote bone repair [53]. FGF4 has been shown to have potential in regulating glucose and lipid metabolism through its central mechanism and AMPK-GLUT4 signaling axis in peripheral skeletal muscle [54, 55]. However, studies on the role of FGF4 in macrophages and spinal cord injury treatment are limited. Consistent with previous studies showing that hydrogel-loaded FGF4 enhanced microtubule stabilization to promote nerve regeneration in the injured spinal cord [56], our findings demonstrate a positive role of FGF4 in axon regeneration targeting synaptic remodeling. Besides, we show that endogenous FGF4 protein is rapidly induced in the injured spinal cord, peaking at 1–3 days post-injury and then declining to near-baseline levels. The exogenous FGF4 supplementation restored and extended this pro-phagocytic signal.

Numerous evidences have shown that FGFR1 and PI3K/AKT pathways affect multiple activities such as macrophage polarization, metabolism, phagocytosis and inflammatory response [57]. In atherosclerosis studies, scavenger receptor Lox-1 activates macrophage FGFR1 to promote lipid uptake, and inhibitor AZD4547 blocks FGFR1 to alleviate the progression of [58]. After FGFR1 activation, it may indirectly regulate the activity of PI3K/AKT pathway through its downstream substrate FRS2 and other adaptor proteins [59]. We found that pharmacological blockade of FGFR1 with PD173074 abrogated FGF4-induced phosphorylation of FGFR1, PI3K and AKT and significantly reduced the formation of myelin debris and lipid droplets in BMDMs. This suggests that FGFR1 activation is essential for FGF4-triggered enhancement of myelin debris uptake. These data further reveal a direct link between FGFR1-PI3K /AKT activation and the phagocytic clearance of myelin debris.

A major challenge after enhanced uptake of myelin debris is the risk of pushing macrophages toward a foamy, pyroptosis-prone state [60]. This shares many features with foam cells in atherosclerotic plaques, including cholesterol crystal formation, lysosomal dysfunction, and activation of the NLRP3 inflammasome [20]. The NLRP3-caspase-1-GSDMD signaling pathway translates the excessive lipid load into IL-1β/IL-18 release and gasdermin-mediated membrane pore formation, which ultimately leads to lytic pyroptosis [61]. For example, trehalose enhanced TFEB activity and nuclear localization, enhanced macrophage autophagy and promoted lipid degradation to reduce foam cell formation [62]. MCC950 (a selective NLRP3 inhibitor) inhibited pyroptosis expression in THP-1-derived foam cells [63]. However, the solutions related to lysosomal function and pyroptosis of foam macrophages in SCI are still limited. Consistent with this paradigm, we observed that myelin debris exposure increased the expression of pyroptosis-related proteins while decreasing lysosomal CTSD, a key protease for lipid degradation. FGF4 treatment reversed these changes in vivo, increasing ABCA1 and CTSD levels, decreasing inflammasome and pyroptosis markers.

The enhancement of phagocytic capacity of macrophages involves phagocytic receptors, internal metabolic state and microenvironmental signals. A variety of receptors have been reported to enhance the phagocytic function, including Toll-like receptor 4, scavenger receptor (SR-A, CD36), complement receptor CR3, Fc receptor, etc [7]. In addition, signaling pathways and metabolic reprogramming also play an important role. Activation of the NF-κB pathway as well as the Irg1/itaconate axis enhances [64, 65]. To further dissect the downstream molecular program engaged by FGF4, we performed RNA-seq on BMDMs treated with myelin debris with or without FGF4. Pathway analysis revealed enrichment of genes involved in immune receptor signaling, protein digestion and absorption, and NF-κB/chemokine pathways rather than canonical phagocytic receptor signatures alone, indicating that FGF4 globally reprograms macrophage activation states.

Based on RNA sequencing, we identified Clec10a as an important effector of FGF4-enhanced phagocytic program. Clec10a (macrophage galactose-type lectin, MGL, CD301) is traditionally characterized as a C-type lectin that binds terminal N-acetyl-galactosamine (GalNAc)–containing glycans and contributes to antigen uptake and tolerance induction in dendritic cells and macrophages [66]. Studies have found that CLEC10A is involved in the development of various diseases. The phagocytic activity of CLEC10A positive macrophages in breast cancer is enhanced to remove damaged cells [67]. CLEC10A also binds to the GalNAc structure of the Staphylococcus aureus cell wall [68]. Based on this classical view, one could hypothesize that Clec10a might recognize GalNAc-containing structures on myelin debris. However, our D-GalNAc competition experiments did not diminish FGF4-induced myelin debris uptake, and 4 °C binding assays showed minimal Clec10a–myelin colocalization at the cell surface. Instead, time-course imaging revealed that Clec10a increasingly colocalized with internalized Dil-labeled myelin debris, closely tracking its transit through endosome–lysosome compartments. These findings suggest that, Clec10a functions predominantly as an intracellular regulator of phagosome maturation or lysosomal processing, rather than as a carbohydrate-recognition receptor for myelin debris binding. One possibility is that Clec10a acts as a scaffolding or signaling component on endolysosomal membranes, coordinating trafficking, acidification, or protease activation; another is that intracellular Clec10a senses glycan changes on myelin-containing vesicles to modulate downstream signaling. These hypotheses warrant direct testing using domain-specific Clec10a mutants, and high-resolution live-cell imaging to resolve Clec10a dynamics during myelin debris trafficking.

It has been shown that macrophages degrade myelin debris mainly through endosome-lysosomes [69]. Co-localization of late endosomes, lysosomes and the myelin protein PLP has been found in macrophages engulfing myelin debris [22], and it has also been suggested that microglia are degraded through the autophagy-lysosomal pathway during aging [70]. Consistent with previous studies, we found that MBP^+^ myelin debris co-localized with Rab7, Lamp1 and CTSD in BMDMs. This supports the idea that myelin debris are primarily degraded through the classical endosomal lysosomal pathway. In addition, FGF4 was found to accelerate endosomal lysosomal transport of myelin debris and improve lysosomal function.

Functional recovery following spinal cord injury is a complex process influenced by various factors, including neuronal survival, axonal remodeling, remyelination, vascular alterations, and additional mechanisms [71]. Targeting the removal of myelin debris helps get around two big problems at once: regeneration inhibition and inflammatory/toxic stress [72]. This greatly increases the chances of SCI repair and functional improvement. In our study, several lines of evidence further support a macrophage-centered interpretation, indicating that enhanced myelin debris clearance is a principal driver of the behavioral benefits observed after FGF4 treatment. First, we assessed the expression dynamics of endogenous FGF4 after injury. FGF4 was relatively enriched in regions of macrophage accumulation at early time points, yet endogenous FGF4 became insufficient during the peak phase of macrophage-mediated myelin debris clearance; importantly, exogenous FGF4 supplementation promoted macrophage-associated myelin debris clearance in vivo. Second, we found that FGF4 alleviates a well-recognized bottleneck in SCI, namely inefficient degradation of myelin debris, by promoting endosome-to-lysosome trafficking of internalized myelin cargo and restoring lysosomal proteolytic capacity. Third, lipid overload and inflammasome activation after SCI, both of which are closely related to chronic inflammation caused by persistent myelin debris, were lessened after FGF4 treatment. Finally, blockade of FGFR1 signaling or silencing of Clec10a weakened the pro-clearance phenotype of macrophages, supporting a pathway-specific mechanism that likely contributes to the in vivo recovery. Overall, these results suggest that the behavioral improvements are best explained by better clearance of myelin debris by macrophages.

Several limitations of this study warrant recognition. First, although we observed an early and transient increase in endogenous FGF4 expression following SCI, the upstream regulatory mechanisms responsible for its induction remain undefined. Second, while loss-of-function experiments demonstrated that Clec10a is required for FGF4-mediated enhancement of myelin debris degradation, the precise molecular mechanisms by which Clec10a functions—particularly its intracellular trafficking behavior, interaction partners, and direct role in phagosome-lysosome fusion were not fully resolved. Third, although our work focuses primarily on macrophages, FGF4 may also influence other cell types expressing FGFR1, including astrocytes, endothelial cells, and neural progenitors, and the potential contribution of these populations to the in vivo therapeutic effects was not investigated. Despite these limitations, the present study provides compelling evidence for FGF4 as a regulator of macrophage-mediated myelin debris clearance and identifies the FGFR1–PI3K/AKT–Clec10a axis as a promising therapeutic pathway for SCI repair.

## Conclusions

This study reveals a previously unrecognized role for FGF4 in regulating macrophage-mediated myelin debris clearance through Clec10a-mediated mechanisms. By restoring lysosomal function, suppressing inflammasome activation, and improving lipid handling, FGF4 transforms macrophages into a pro-resolving phenotype that promotes neuronal protection and functional recovery following SCI. These findings highlight the FGF4–Clec10a axis as a promising therapeutic target for CNS injuries characterized by defective debris clearance and chronic inflammation.

## Supplementary Information

Supplementary Material 1.

## Data Availability

The RNA-sequencing data generated in this study have been deposited in the NCBI Sequence Read Archive (SRA) under BioProject accession number PRJNA1379301. All other data supporting the findings of this study are available from the corresponding author upon reasonable request.

## Abbreviations

SCI: Spinal Cord Injury
FGF4: Fibroblast Growth Factor 4
Clec10a: C-type Lectin Domain Family 10 Member A
MBP: Myelin Basic Protein
OMgp: Oligodendrocyte Myelin Glycoprotein
MD: Myelin Debris
TLR4: Toll-Like Receptor 4
MSR1: Macrophage Scavenger Receptor 1
ABCA1: ATP-Binding Cassette Transporter A1
NLRP3: NOD-Like Receptor Thermal Protein Domain Associated Protein 3
GSDMD: Gasdermin D
Lamp1: Lysosomal Associated Membrane Protein 1
CTSD: Cathepsin D
BMDMs: Bone Marrow-Derived Macrophages
MoDMs: Monocyte-derived macrophages
FBS: Fetal Bovine Serum
D-GalNAc: N-acetyl-D-galactosamine
HBSS: Hank’s Balanced Salt Solution
HE: Hematoxylin and Eosin
LFB: Luxol Fast Blue
HRP: Horseradish Peroxidase
DAB: Diaminobenzidine
TEM: Transmission Electron Microscopy
LMP: Lysosomal Membrane Permeabilization
BMS: Basso Mouse Scale
MEPs: Motor Evoked Potentials
Fcgr4: Fc gamma receptor 4
Cd163: Cluster of Differentiation 163
Irf4: Interferon regulatory factor 4
Ccl6: C-C motif chemokine ligand 6
Scd1: Stearoyl-CoA desaturase 1
CD86: Cluster of Differentiation 86
CD206: Cluster of Differentiation 206
