# Supplementary material for: FGF4 activates FGFR1 - PI3K/AKT signaling to enhance Clec10a-mediated intracellular myelin debris processing and promote spinal cord repair
Source: J Neuroinflammation. 2026 Feb 23;23:107. doi: 10.1186/s12974-026-03743-0 (PMC13037223; doi:10.1186/s12974-026-03743-0)
Supplement: Supplementary file 1 — Supplementary Material 1. [file 12974_2026_3743_MOESM1_ESM.docx]

**
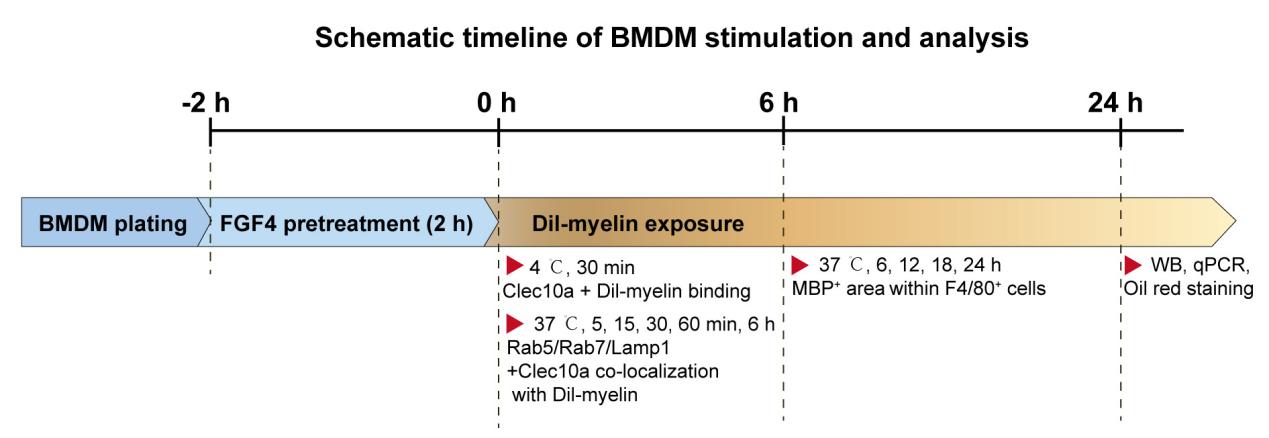
**

**Supplementary Fig. S1** Schematic timeline of BMDM stimulation and downstream assays. BMDMs were plated and pretreated with FGF4 for 2 h prior to myelin debirs exposure. At 0 h, Dil-labeled myelin debris was added to initiate exposure. For binding assays, cells were incubated with Dil-myelin at 4 °C for 30 min and Clec10a–myelin association was assessed. For short-term trafficking assays, cells were incubated at 37 °C and analyzed at 5, 15, 30, and 60 min and 6 h to evaluate colocalization of internalized Dil-myelin with Rab5, Rab7, Lamp1, and Clec10a. For longer-term readouts, MBP⁺ area within F4/80⁺ cells was quantified at 37 °C at 6, 12, 18, and 24 h after myelin exposure. At 24 h, cells were collected for WB, qPCR, and Oil Red O staining as indicated.

**
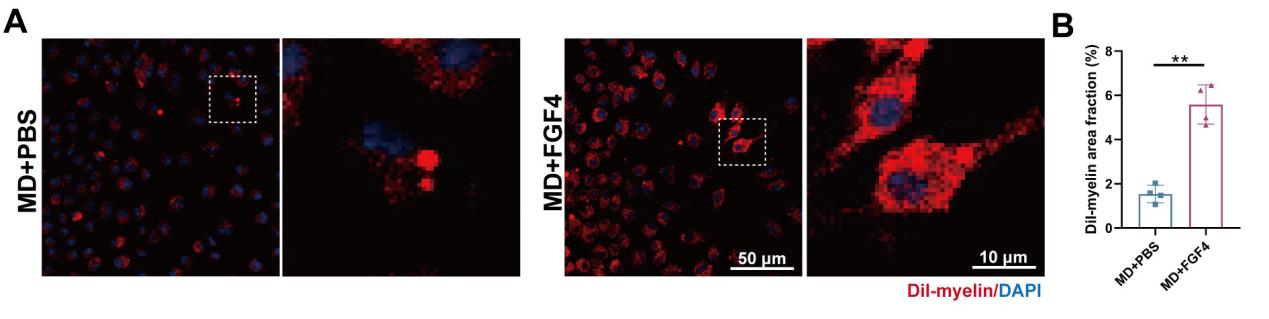
**

**Supplementary Fig. S2** FGF4 promotes early internalization of Dil-labeled myelin debris by macrophages. **A** Representative fluorescence images of BMDMs incubated with Dil-myelin (red) under the indicated conditions (MD+PBS and MD+FGF4). Nuclei are stained with DAPI (blue). Boxed regions are shown at higher magnification to illustrate intracellular Dil-myelin signal. Scale bars: 50 µm, 10 µm (magnified). **B** Quantification of Dil-myelin area fraction (%) within the predefined region of interest (ROI) at 37 °C for 60 min under the indicated conditions (n = 4 wells). **p < 0.01.

**
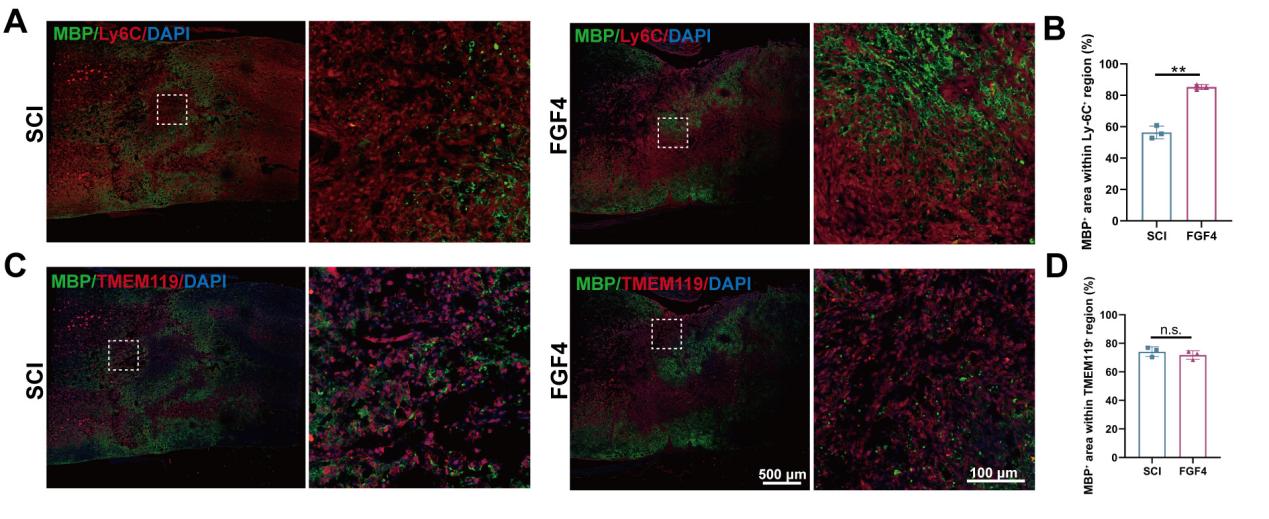
**

**Supplementary Fig. S3** FGF4 preferentially enhances myelin debris uptake in infiltrating Ly6C⁺ monocyte-derived macrophages (MoDMs) rather than resident TMEM119⁺ microglia in vivo. **A** Representative immunofluorescence images of injured spinal cord sections stained for MBP (green), Ly6C (red), and DAPI (blue) in SCI and FGF4 groups at 7 dpi. The boxed regions magnified in the corresponding right panels. Scale bars: 500 µm, 100 µm (magnified). **B** Quantification of MBP⁺ area within the Ly6C⁺ region (%) in SCI and FGF4 groups (n = 3 animals). **C** Representative immunofluorescence images of injured spinal cord sections stained for MBP (green), TMEM119 (red), and DAPI (blue) in SCI and FGF4 groups at 7 dpi. The boxed regions magnified in the corresponding right panels. Scale bars: 500 µm, 100 µm (magnified). **D** Quantification of MBP⁺ area within the TMEM119⁺ region (%) in SCI and FGF4 groups (n = 3 animals). **p < 0.01, n.s. = not significant.

**
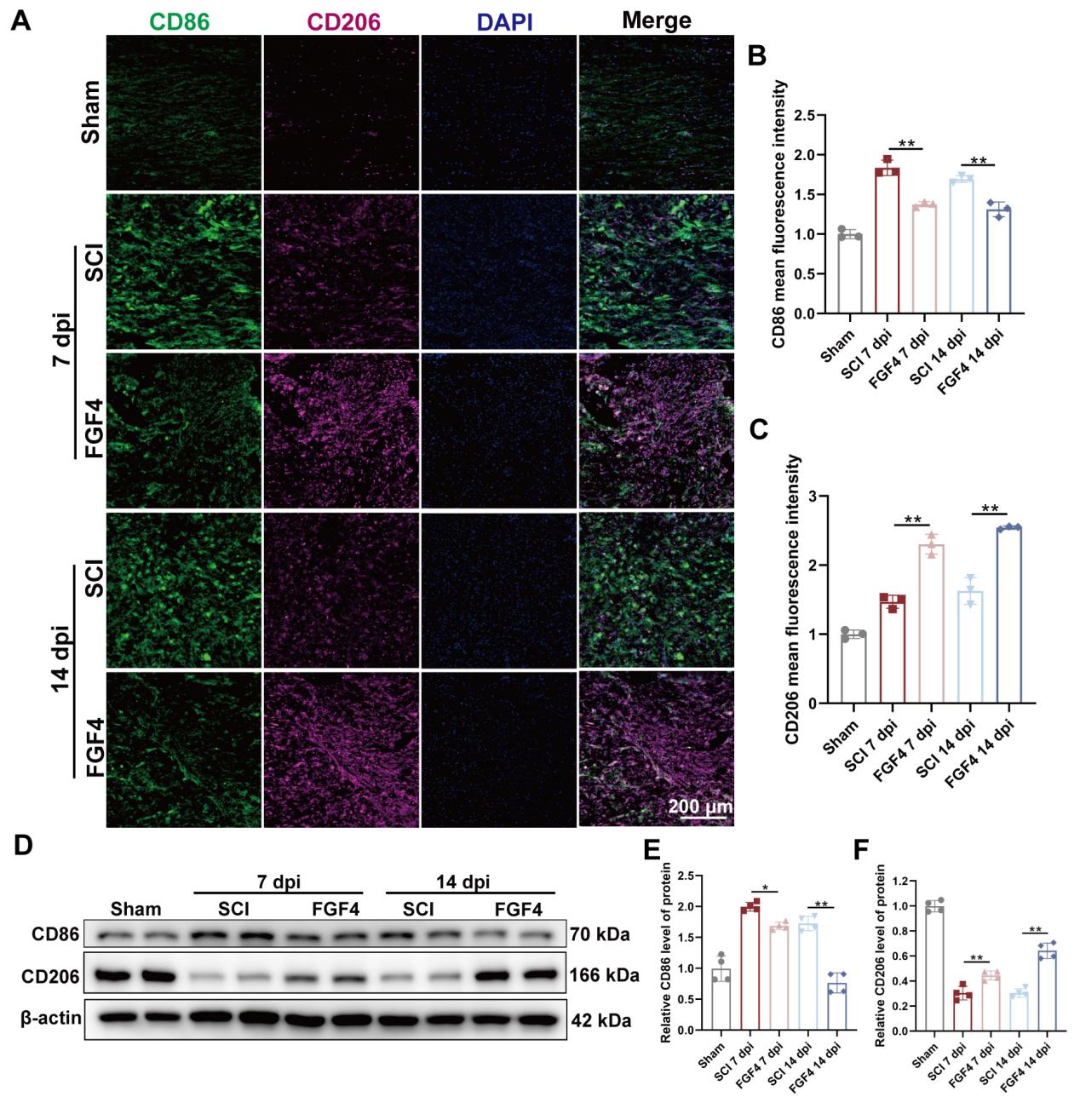
Supplementary Fig. S4** FGF4 shifts macrophage polarization toward an anti-inflammatory, pro-repair phenotype in vivo. **A** Representative immunofluorescence images of spinal cord sections at the lesion epicenter from sham-operated mice and mice subjected to SCI with or without FGF4 treatment, assessed at 7 and 14 dpi. Sections were stained for the M1 macrophage marker CD86 (green), the M2 macrophage marker CD206 (purple), and nuclei (DAPI, blue). Scale bar: 200 µm. **B** Quantitative analysis of the mean fluorescence intensity of CD86 (n = 3 animals). **C** Quantitative analysis of the mean fluorescence intensity of CD206 (n = 3 animals). **D** Representative Western blot images showing the protein levels of CD86 and CD206 in spinal cord tissue lysates from the Sham, SCI, and FGF4 groups at 7 and 14 dpi. β-actin was used as a loading control. **E** Densitometric quantification of relative CD86 protein levels normalized to β-actin (n = 4 animals). **F** Densitometric quantification of relative CD206 protein levels normalized to β-actin (n = 4 animals). *p < 0.05, **p < 0.01.

**
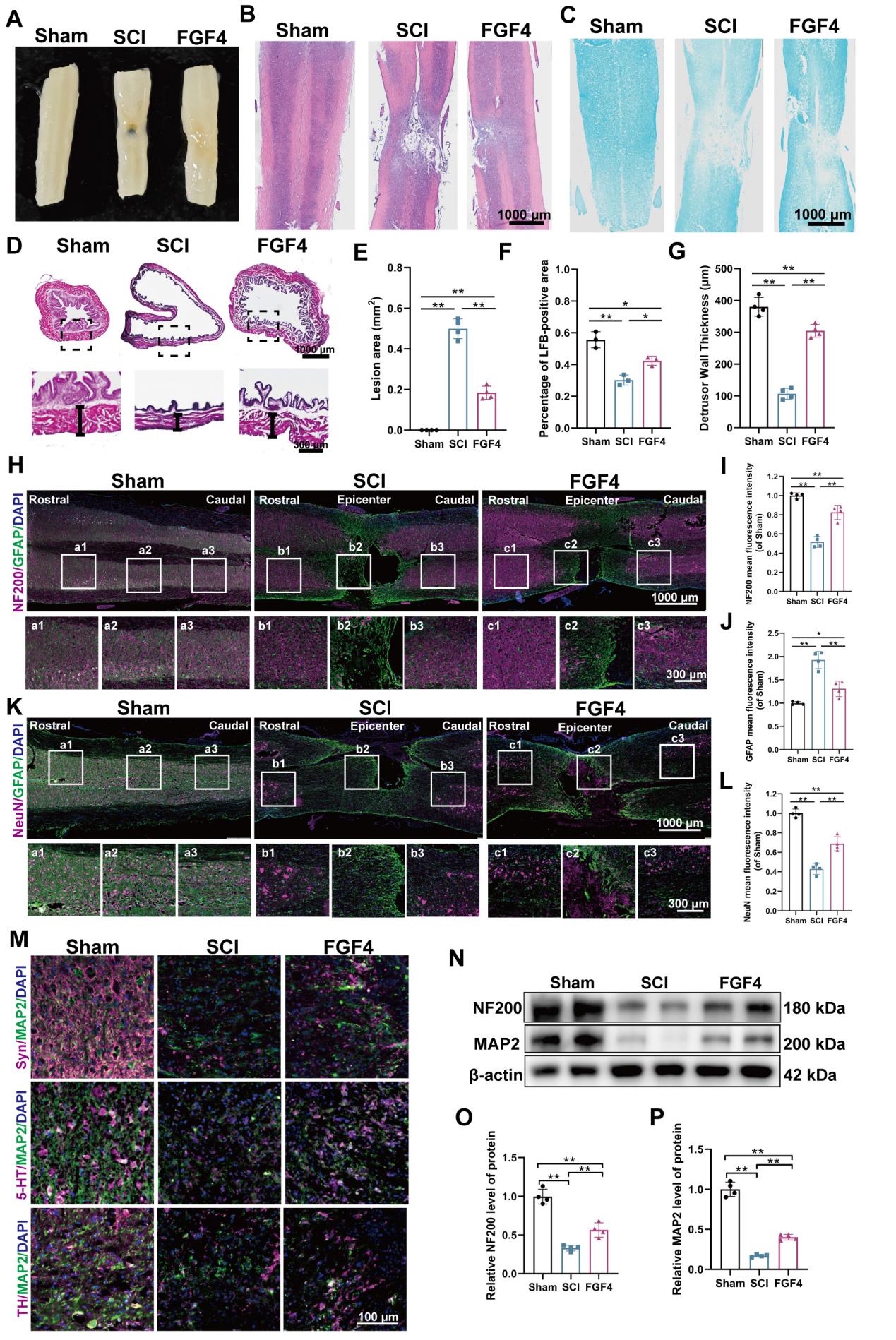
**

**Supplementary Fig. S5** FGF4 promotes long-term structural repair of the injured spinal cord. **A** Representative macroscopic images of spinal cords from Sham, SCI, and FGF4 groups at 42 dpi, showing the overall lesion appearance. **B** Hematoxylin and Eosin (H&E) staining of spinal cord sections at the lesion epicenter from the indicated groups at 42 dpi. Scale bar: 1000 µm. **C** Luxol Fast Blue (LFB) staining for myelin in spinal cord sections from the indicated groups at 42 dpi. Scale bar: 1000 µm. **D** H&E staining of bladder tissue sections from the indicated groups at 42 dpi. Lower panels show higher-magnification views of the boxed regions, highlighting the detrusor muscle layer. Scale bars: 1000 µm, 300 µm (magnified). **E** Quantification of the lesion area (mm²) from H&E-stained spinal cord sections in **(B)** (n = 4 animals). **F**​ Quantitative analysis of the percentage of LFB-positive area in the spinal cord from images in **(C)** (n = 3 animals). **G** Measurement of detrusor wall thickness (µm) from bladder tissue sections in **(D)** (n = 4 animals). **H** Representative immunofluorescence images of spinal cord sections stained for neurofilament-200 (NF200, purple) and glial fibrillary acidic protein (GFAP, green). Nuclei are stained with DAPI (blue). Magnified views of the boxed areas **(a1-a3, b1-b3, c1-c3)** in the rostral, epicenter, and caudal regions are shown below. Scale bars: 1000 µm, 300 µm (magnified). **I** Quantitative analysis of the mean fluorescence intensity of NF200 (n = 4 animals). **J** Quantitative analysis of the mean fluorescence intensity of GFAP (n = 4 animals). **K** Representative immunofluorescence images of spinal cord sections stained for the neuronal marker NeuN (purple) and GFAP (green). Nuclei are stained with DAPI (blue). Boxed regions are magnified below. Scale bars: 1000 µm, 300 µm (magnified). **L** Quantitative analysis of the mean fluorescence intensity of NeuN (n = 4 animals). **M** Representative immunofluorescence images of the spinal cord lesion area at 42 dpi, showing co-staining for synapsin (Syn, purple) and microtubule-associated protein 2 (MAP2, green); 5-hydroxytryptamine (5-HT, purple) and MAP2 (green); and tyrosine hydroxylase (TH, purple) and MAP2 (green). Nuclei are stained with DAPI (blue). Scale bar: 100 µm. **N** Representative Western blot images of NF200 and MAP2 protein expression in spinal cord tissues from the indicated groups at 42 dpi. β-actin served as a loading control. **O** Densitometric quantification of relative NF200 protein levels normalized to β-actin (n = 4 animals). **P** Densitometric quantification of relative MAP2 protein levels normalized to β-actin (n = 4 animals). *p < 0.05, **p < 0.01.

**
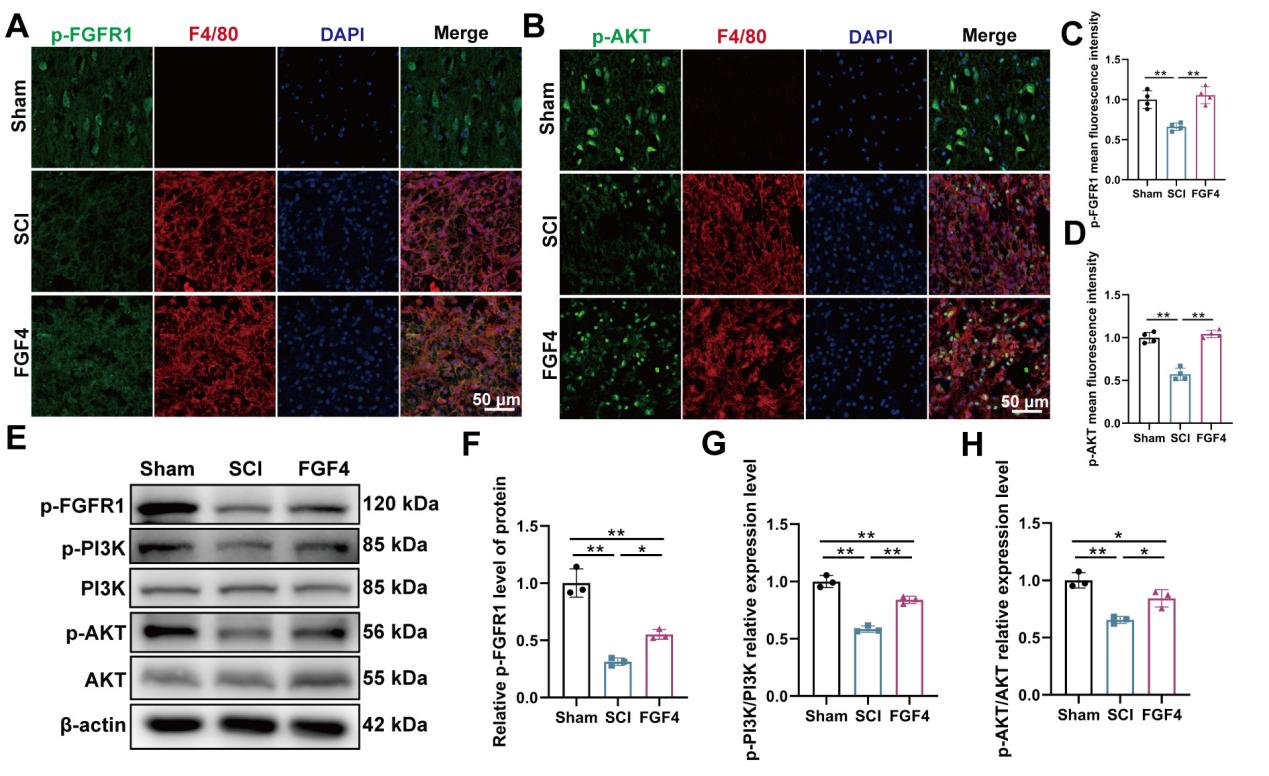
**

**Supplementary Fig. S6** In vivo target engagement of the FGFR1–PI3K/AKT pathway in lesion-associated macrophages after FGF4 administration. **A** Representative immunofluorescence images of injured spinal cord sections (7 dpi) stained for p-FGFR1 (green), F4/80 (red), and DAPI (blue) in Sham, SCI, and FGF4 groups. Scale bar: 50 µm. **B** Representative immunofluorescence images (7 dpi) stained for p-AKT (green), F4/80 (red), and DAPI (blue) in Sham, SCI, and FGF4 groups. Scale bar: 50 µm. **C** Quantification of p-FGFR1 mean fluorescence intensity (n = 3 animals). **D** Quantification of p-AKT mean fluorescence intensity (n = 3 animals). **E** Representative Western blots images of p-FGFR1, p-PI3K, PI3K, p-AKT, AKT, and β-actin in injured spinal cord tissue lysates from Sham, SCI, and FGF4 groups (7 dpi). **F–H** Densitometric quantification of p-FGFR1 **(F)**, p-PI3K/PI3K **(G)**, and p-AKT/AKT **(H)** (n = 3 animals). *p < 0.05, **p < 0.01.


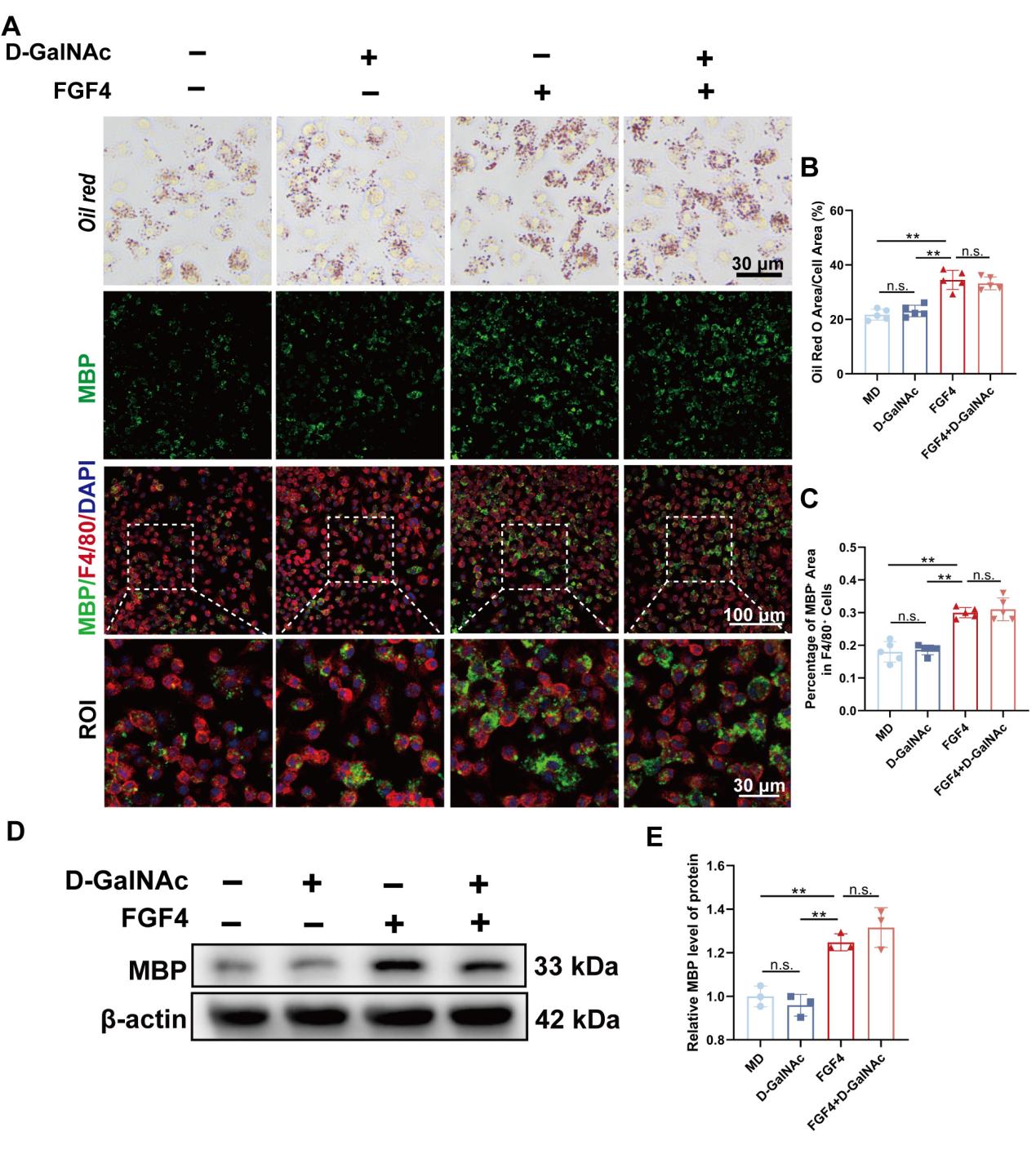


**Supplementary Fig. S7** D-GalNAc competitive inhibition experiment reveals that FGF4 does not interact with Clec10a through classical carbohydrate-binding competition. **A** Representative Oil Red O staining of BMDMs following treatment with D-GalNAC (a ligand of Clec10a) as a carbohydrate competitor. Scale bar: 30 µm. Representative immunofluorescence images of BMDMs stained for internalized myelin debris (MBP, green), macrophages (F4/80, red), and nuclei (DAPI, blue). Boxed regions are magnified below. Scale bars: 100 µm, 30 µm (magnified). **B** Quantitative analysis of the Oil Red O-positive area expressed as a percentage (%) of the total cell area (n = 5 wells). **C** Quantitative analysis of the percentage of MBP^+^ area within F4/80^+^ cells (n = 5 wells). **D** Representative Western blot images of MBP protein expression in BMDM lysates under D-GalNAC treatment. β-actin was used as a loading control. **E** Densitometric quantification of relative MBP protein levels normalized to β-actin (n = 3 wells). **p < 0.01, n.s. = not significant.


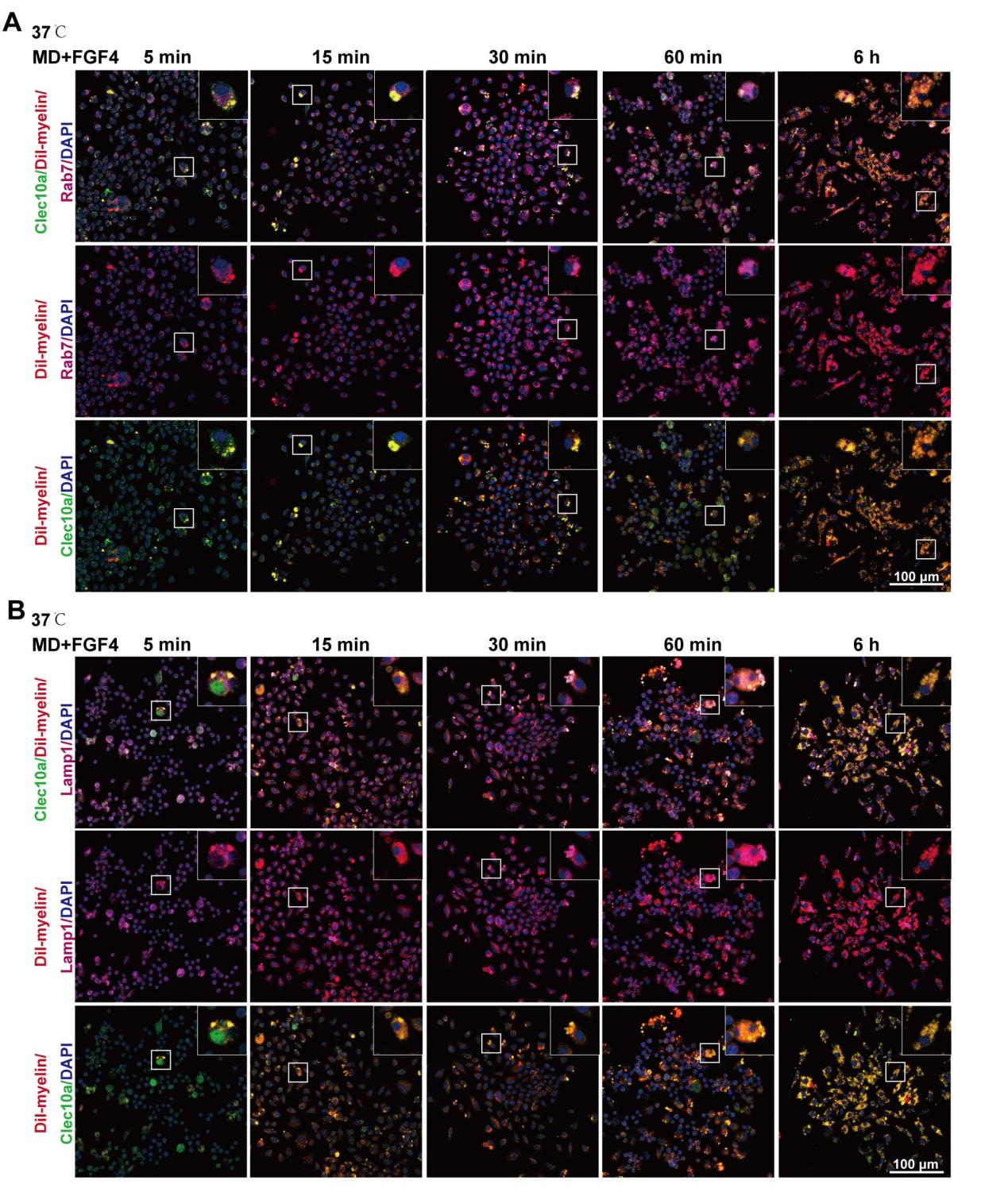


**Supplementary Fig. S8** Time-dependent intracellular trafficking of myelin debris in FGF4-treated macrophages. **A** Representative immunofluorescence images of BMDMs pretreated with FGF4 for 2 h and subsequently incubated with Dil-labeled myelin debris (red) at 37 °C for 5, 15, 30, 60 min, and 6 h. Cells were immunostained for Clec10a (green), Rab7 (purple), and DAPI (blue) to visualize the temporal progression of internalized myelin toward late endosomal compartments. Scale bar: 100 μm. **B** Representative immunofluorescence images of BMDMs pretreated with FGF4 for 2 h and subsequently incubated with Dil-labeled myelin debris (red) at 37 °C for 5, 15, 30, 60 min, and 6 h. Cells were immunostained for Clec10a (green), Lamp1 (purple), and DAPI (blue) to track lysosomal trafficking of internalized myelin over time. Scale bar: 100 μm.


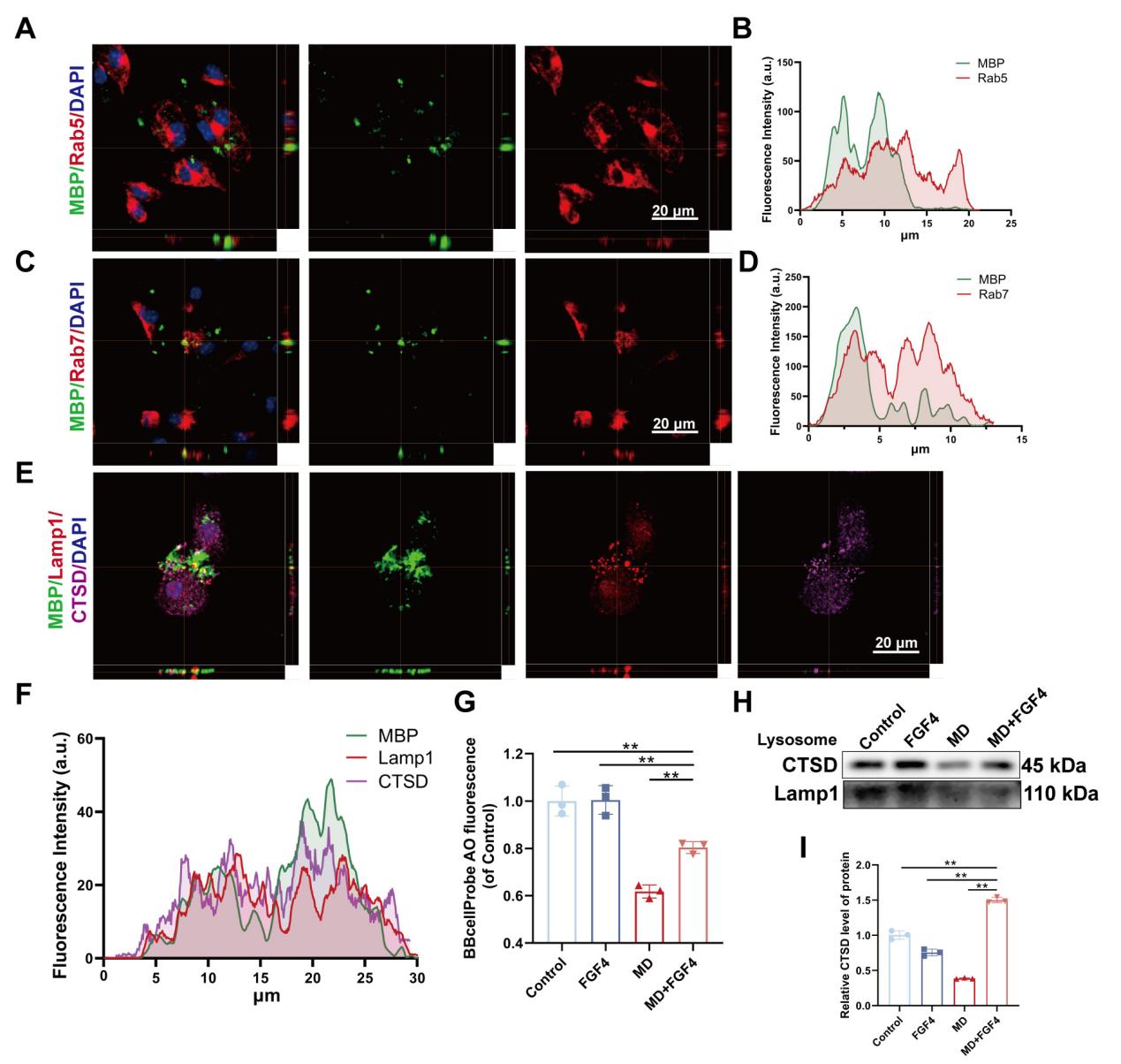


**Supplementary Fig. S9** Analysis of co-localization of internalized myelin debris with Rab5, Rab7, Lamp1, and CTSD, and lysosomal functional changes in macrophages. **A** Representative confocal Z-stack images of macrophages stained for MBP (green), Rab5 (red) and DAPI (blue) at 18 h after myelin exposure. Orthogonal XZ and YZ projections show the three-dimensional distribution of MBP-positive myelin debris relative to Rab5. Scale bar: 20 µm. **B** Line-scan fluorescence intensity profile illustrating the spatial distribution of MBP and Rab5 signals along the indicated axis. **C** Confocal Z-stack images of macrophages stained for MBP (green), Rab7 (red) and DAPI (blue) at 18 h after myelin exposure, with orthogonal projections showing the localization of internalized myelin relative to Rab7. Scale bar: 20 µm. **D** Line-scan fluorescence intensity profile of MBP and Rab7 corresponding to panel **(C)**. **E** Confocal Z-stack images of macrophages stained for MBP (green), Lamp1 (red), CTSD (purple) and DAPI (blue) at 18 h after myelin exposure. Orthogonal views display the spatial distribution of myelin debris within Lamp1- and CTSD-positive compartments. Scale bar: 20 µm. **F** Line-scan fluorescence intensity profile showing MBP, Lamp1, and CTSD signal distribution along the indicated axis. **G** Quantification of BBcellProbe AO fluorescence (of Control) used for the assessment of lysosomal membrane permeabilization (n = 3 wells). **H** Representative Western blot images of cathepsin D (CTSD) protein expression in purified lysosomal fractions isolated from BMDMs. Lysosomal-associated membrane protein 1 (Lamp1) was used as a lysosomal loading control. **I** Quantitative analysis of relative CTSD protein levels in the lysosomal fractions, normalized to Lamp1 (n = 3 wells). **p < 0.01.

**
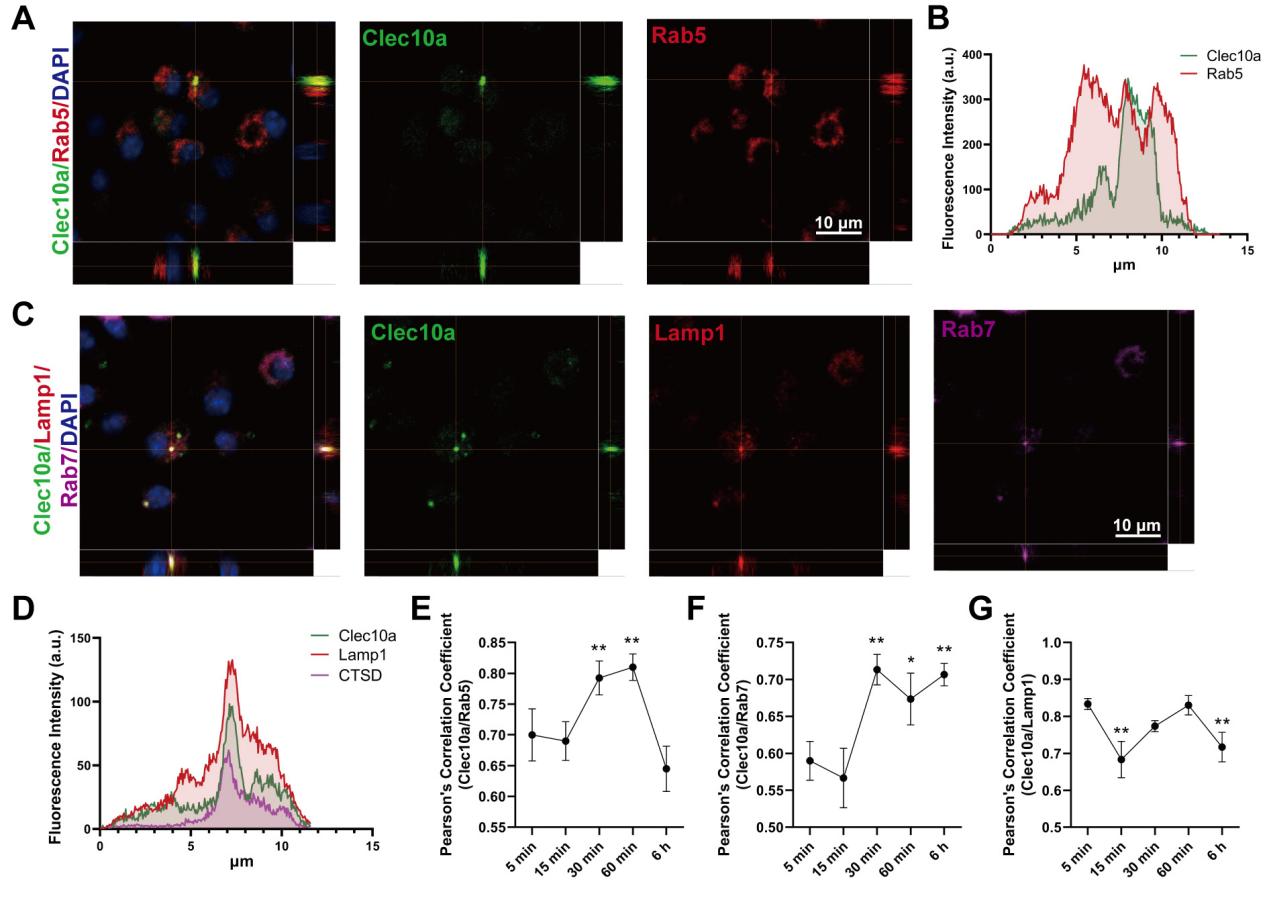
**

**Supplementary Fig. S10** Dynamic endolysosomal localization of Clec10a during myelin debris trafficking in macrophages. **A** Representative confocal Z-stack images of macrophages stained for Clec10a (green), Rab5 (red), and DAPI (blue). Orthogonal XZ and YZ projections show the three-dimensional distribution of Clec10a relative to Rab5. Scale bar: 10 µm. **B** Line-scan fluorescence intensity profile illustrating the spatial distribution of Clec10a and Rab5 signals along the indicated axis in panel. **C** Representative confocal Z-stack images of macrophages stained for Clec10a (green), Lamp1 (red), Rab7 (purple), and DAPI (blue). Orthogonal projections show the localization of Clec10a relative to Lamp1- and Rab7-positive compartments. Scale bar: 10 µm. **D** Line-scan fluorescence intensity profile showing the spatial distribution of Clec10a, Lamp1, and Rab7 signals along the indicated axis in panel. **E–G** Quantitative analysis of Pearson’s correlation coefficients for Clec10a colocalization with Rab5 **(E)**, Rab7 **(F)**, and Lamp1 **(G)** at the indicated time points after myelin exposure (n = 3 wells). *p < 0.05, **p < 0.01.

**
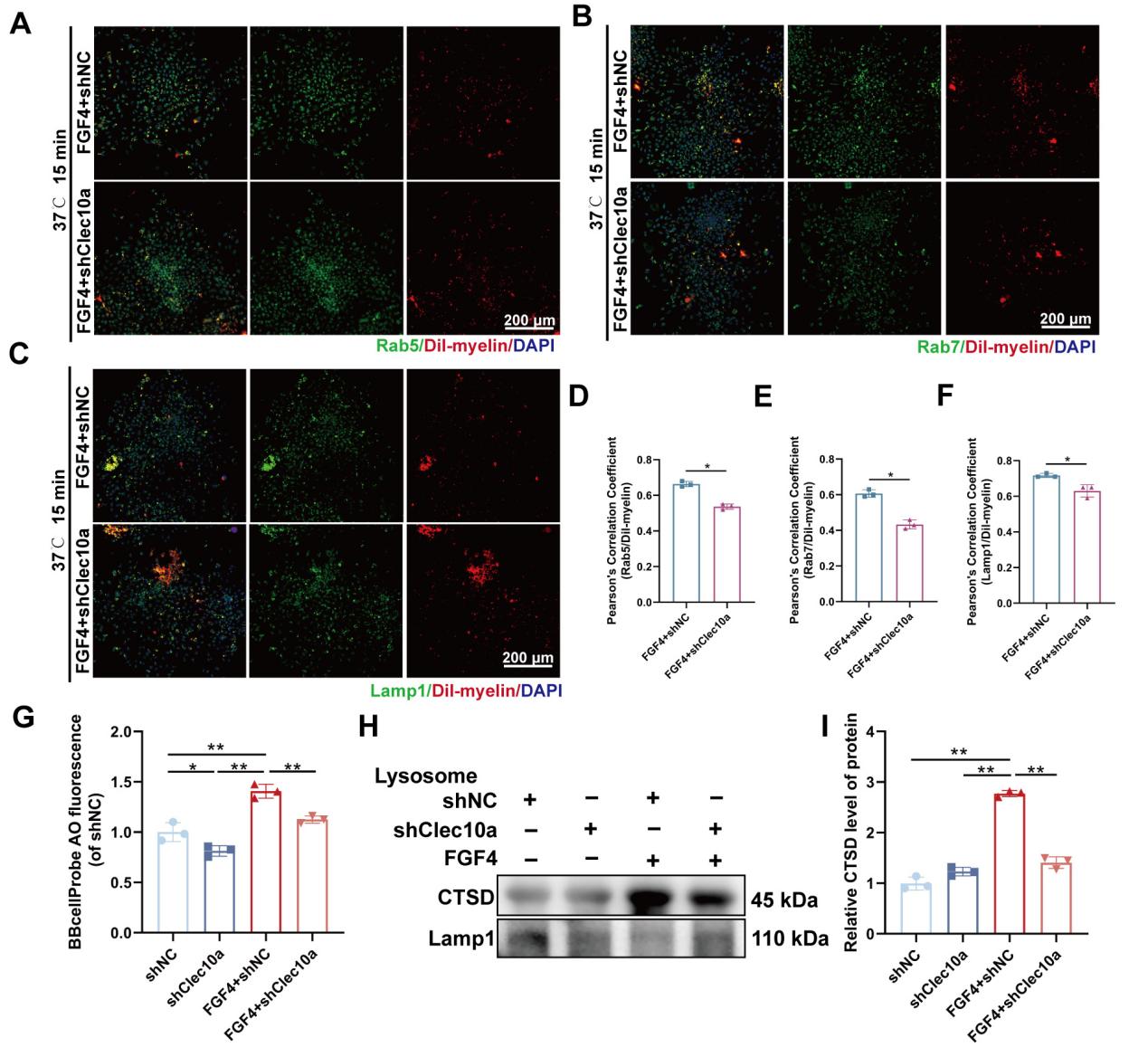
**

**Supplementary Fig. S11** Clec10a knockdown impairs FGF4-promoted endolysosomal trafficking and lysosomal function in macrophages. **A–C** Representative immunofluorescence images showing colocalization of Dil-myelin (red) with Rab5 (green) **(A)**, Rab7 (green) **(B)**, or Lamp1 (green) **(C)** in BMDMs treated with FGF4 and transduced with shNC or shClec10a, followed by incubation with myelin debris at 37 °C for 15 min. Nuclei are stained with DAPI (blue). Scale bars: 200 µm. **D–F** Quantitative analysis of Pearson’s correlation coefficients for Rab5/Dil-myelin **(D)**, Rab7/Dil-myelin **(E)**, and Lamp1/Dil-myelin **(F)** colocalization (n = 3 wells). **G** Quantification of BBcellProbe AO fluorescence intensity to assess lysosomal membrane permeabilization (n = 3 wells). **H** Representative Western blot images of CTSD in purified lysosomal fractions isolated from BMDMs under the indicated conditions, Lamp1 was used as a lysosomal loading control. **I** Densitometric quantification of relative CTSD levels in lysosomal fractions, normalized to Lamp1 (n = 3 wells). *p < 0.05, **p < 0.01.

**Supplementary Table S1**

Primer sequences used in this study.

| **Gene** | **Forward primer** | **Reverse primer** |
| --- | --- | --- |
| Fcgr4 | CCACCGTGGCATCAAATCAC | GTCCTGAGGTTCCTTGCTCC |
| Cd163 | GATGATTCTCTCCCTCCGTGG | GTGTCCACCCATTCCAAAGC |
| Irf4 | AATGGTTGCCAGGTGACAGG | TGCTTGGCTCAATGGGGATT |
| Ccl6 | GTTCGCCCTGCCACAATAGA | AATTTCACCCCAAGAGCCCA |
| Scd1 | TGGAGACGGGAGTCACAAGA | ACACCCCGATAGCAATATCCAG |
| Clec10a | CCCCACTGCAGCCAGATAAC | CACAGATCCAGCGGAAGGTT |
| β-actin | CACTGTCGAGTCGCGTCC | CGCAGCGATATCGTCATCCA |
